# Direct economic burden of acute coronary syndromes in the Portuguese National Health Service—facts and trends between 2002 and 2022

**DOI:** 10.3389/fpubh.2025.1433307

**Published:** 2025-03-18

**Authors:** Francisco Madeira, Carla Martins, Susana Viegas, Ana Teresa Timóteo, Fátima Loureiro, Julian Perelman

**Affiliations:** ^1^NOVA National School of Public Health, Public Health Research Centre, Comprehensive Health Research Center, CHRC, REAL, CCAL, NOVA University Lisbon, Lisbon, Portugal; ^2^Cardiology Department, Santa Marta Hospital, Centro Hospitalar Universitário Lisboa Central, Lisbon, Portugal; ^3^NOVA Medical School, Comprehensive Health Research Center (CHRC), NOVA University Lisbon, Lisbon, Portugal; ^4^National Center for Data Collection in Cardiology, Portuguese Society of Cardiology, Coimbra, Portugal

**Keywords:** acute coronary syndrome, acute myocadial infarction, cost analysis, economic burden of disease, Portugal

## Abstract

**Aim:**

We estimated the average direct cost per Acute Coronary Syndromes (ACS) in-patient episodes by diagnosis, namely ST-elevation myocardial infarction (STEMI), non-ST elevation myocardial infarction (NSTEMI), unstable angina (UA), and undetermined AMI (Acute Myocardial Infarction). We also analyzed the changes in direct costs over time between 2002 and 2022, and the total direct economic burden of ACS hospitalizations for the Portuguese National Health Service (NHS).

**Methods:**

We used the Portuguese Registry of Acute Coronary Syndromes (61,440 ACS hospitalizations), a cohort of people with ACS, recruited and followed from first hospitalization. A direct cost analysis was conducted. As resources, we considered health professional working hours, non-medical resources used during in-patient stays, laboratory and diagnostic tests, interventional cardiology procedures, pharmaceuticals, hospitalization-related complications, rehabilitation services, and death costs. A multivariate analysis was performed to identify the main cost determinants.

**Results:**

The average cost per ACS patient from 2002 to 2022 was 6,280.79 €. A significantly higher average cost was observed among patients diagnosed with STEMI of 3,853.26€ (95% confidence interval [CI] 3,690.87 to 4,015.65€), among NSTEMI patients of 1,308.91 € (95% CI 1,173.52 € to 1,444.30 €), and among patients who died during the hospitalization of 12,017.64€ (95% CI 11,232.21 € to 12,803.08 €). Over time, cost trends fluctuated, increasing until 2011 and then gradually decreasing until 2022, apart from 2020. Considering the total universe of 294,307 ACS-hospitalizations, the Portuguese NHS incurred a direct economic burden of 1,831 million euros over the complete period, with total annual costs averaging 87,203,851 €, representing on average 0.93% of the NHS annual health expenditure.

**Conclusion:**

ACS represent a significant direct cost and economic burden for the NHS.

## 1 Introduction

For the last six decades, cardiovascular diseases (CVD) have been the leading cause of mortality in Portugal, accounting for 25.9% (34,452) of all fatalities in 2021 according to the latest published information (1).

Among CVD, Acute Coronary Syndromes (ACS), comprising ST-elevation myocardial infarction (STEMI), non-ST elevation myocardial infarction (NSTEMI) and unstable angina (UA), have a high incidence in the Portuguese population, estimated by us at 134.04 ACS cases per 100,000 inhabitants for the last 21 years (unpublished data).

Regarding acute myocardial infarction (AMI), the inpatient hospital discharges (including subsequent myocardial infarction) for 2021 in Portugal was 116.6 cases per 100,000 inhabitants, below the average for the 27 member states of the European Union of 168.58 cases per 100,000 inhabitants (2). Additionally, Portuguese standardised death rate for 2020 was 34.3, close to the European rate of 37.71 cases per 100,000 inhabitants (3). To mitigate these challenges, diagnostic and treatment strategies have evolved over time via the adoption of early coronary reperfusion strategies, developments in pharmacological treatment, enhanced and standardized care and targeting of vulnerable subgroups. While these new techniques have improved prognosis and health outcomes, they have also led to increased costs (4). In a context of tight public health budgets and weak economic growth, as currently faced by Portugal, evaluating these costs and its evolution is essential, to confirm that they are acceptable in the light of the obtained health gains.

Indeed, considering that Portugal ranks as one of the lowest income nations in Europe and faces major state budget constraints, a proper resource allocation is of the utmost importance within a context of low economic growth and restricted state funding. The first step of an accurate resource allocation is the quantification of the disease treatment costs and of its economic burden.

Also, as the Portuguese demographic pyramid inverts, leading to a rapidly growing population ageing, an increase in ACS cases is expected. This is supported by the fact that the ageing ratio in Portugal for 2022 (the most recent year available) is 186 older adult people per 100 young people, with projections to reach 300 older adult people by 2080 (5, 6). The literature has also described the association between the risk of an ACS event and normal cardiovascular ageing (7).

The international literature states that the main costs and resource used by patients with ACS occur in the first year of hospitalization. A Spanish study from the public perspective concluded that the average direct cost per patient with an ACS was 12,252.9 €, with STEMI costing 12,245.7 € and NSTEMI 12,264.7 € (8). As for Italy, the estimated average direct cost from two distinct National Health Service (NHS) areas was 11,464 € (9) and 14,111€ (10), with the first study reporting a cost of 12,112 € for men and 10,307 € for women, and the second a cost of 14,984 € for STEMI, 14,984.5 € for NSTEMI and 12,481.5 € for UA. A Swedish study concluded that the average direct cost per patient from the AMI event up to a 6-year follow-up was approximately 20,000 €, with the highest consumption and costs occurring in the first year, i.e., 12,460 €, up to 90% due to hospitalizations (11). A Canadian study stated a total cost of 19,842 $ per patient in the first year, with the main proportion of this costs attributed to hospitalizations during that first year (80%). The authors also reported that in the first year, STEMI patients were more costly to manage than NSTEMI (21,060 € vs. 19,648 €), even though the last consumed more resources, mainly due to a higher incidence of events (12). Regarding the United States, the average direct medical expenditure per patient was 18,739 $ (13).

The purpose of this study is to estimate the average direct individual cost per ACS episode (and its diagnosis) in the Portuguese NHS, its evolution over the 2002 and 2022 period, and to estimate the total direct economic burden of ACS hospitalizations in the NHS.

Note that this is the first study conducted in Portugal to estimate the direct economic impact of ACS. Although indirect costs may represent a considerable burden due to complications necessitating extensive treatment, the objective of this study was to focus exclusively on the direct cost burden, as the total indirect cost of AMI within the first year after hospitalization has already been estimated at just over 10 million euros, an unexpectedly low value (14).

Furthermore, in light of the expected increase in the number of cases of cardiovascular disease (the most prevalent pathology in Portugal), related with a dramatically ageing population, the disease burden associated with ACS is expected to increase, resulting in an inevitable higher economic burden for the Portuguese NHS. By examining the cost trends associated with this disease and its respective treatment cost drivers, this study provides detailed information on how costs are increasing. These findings provide stakeholders with the necessary data to develop a comprehensive understanding of the underlying causes, which we also elucidate, thereby enabling the elaboration of cost-containment policies to help control public expenditure burdens on the national budget.

## 2 Materials and methods

A direct cost analysis was conducted. This analysis is described by two phases, the first being the identification of resources in physical units and the second the determination of unit values (the valorisation of the resources) (15).

For each resource used, a unit cost (euros) was allocated, to establish the estimated annual healthcare cost per patient with an ACS episode within the first hospitalization.

### 2.1 Sample

We used data from the Portuguese Registry of Acute Coronary Syndromes (ProACS), a cohort of individuals with ACS, recruited and followed from first hospitalization. This cohort was started in 2002, sponsored by the Portuguese Society of Cardiology (SPC), and coordinated by the National Center for Data Collection in Cardiology (CNCDC) (16).The ProACS database comprises detailed information collected throughout an ACS patient’s hospitalisation, including demographic and baseline characteristics, diagnostic and therapeutic data (medication and intervention), and follow-up data at discharge.

The data represents a sample of Portuguese hospitals with cardiology departments or services, whose participation is voluntary. Note that the sample does not cover all hospitals and has been decreasing throughout the years (Supplementary Figure 1); hence, it does not represent all ACS cases in Portugal.

After patients’ selection according to pre-established inclusion or exclusion criteria, data entry is conducted digitally and validated, initially by an automated audit to identify any potential irregularities, and then manually by an investigator (17).

All information is centralized at the CNCDC in Coimbra in an anonymized database. Consequently, the complete analysis was conducted exclusively on site. Only the outputs generated and authorized were collected.

This research was conducted as part of a scientific project, for which access to the ProACS database was requested and approved by the CNCDC of the SPC. The ProACS registry is approved by the Portuguese Data Protection Authority (no. 3140/2010), registered at ClinicalTrials.gov (NCT01642329), and overseen by an Executive Committee (17).

As this study aims to assess the evolution of costs over time, cases whose year of admission was unknown were not considered for analysis. Given that the follow-up of each adult patient (≥18 years) included in ProACS was conducted up to 1 year since the date of hospital admission, cases who did not fulfil these criteria were excluded. So, the final sample included 61,440 cases, with the exclusion of 13,553 cases (18.07%).

### 2.2 Resource use

The resources used have been organised into cost categories, with their constituent variables established in accordance with ProACS.

#### 2.2.1 Inpatient days

The length of stay (LOS) in the hospital was measured according to the number of days spent in hospital. For patients for whom the date of hospital admission and the date of discharge or date of death were present, the LOS was calculated directly through the difference between the date of discharge or of death and the date of hospital admission. If patients did not have this information available, their LOS was attributed following a hierarchical logic, with specific conditions taking precedence over others. If patients had their admission diagnosis and admission year available (derived from the date of hospital admission), we attributed the average LOS of people with the same admission diagnosis and admission year, considering their vital status upon hospital discharge (alive or dead). If no vital status was available, the average LOS of the previous two was assigned. In case the patients only had their admission year available, their LOS was calculated by the average LOS for their admission year, considering their vital status upon hospital discharge (alive or dead). Again, if no vital status was available, the average LOS of the previous two was assigned. The measured LOS are presented in Supplementary Figure 2.

#### 2.2.2 Operating room

Operating room was considered if there was an indication of its use for interventional cardiology procedures.

#### 2.2.3 Medical transport

The medical transport considered was either by an emergency medical ambulance (AEM), by a vehicle for medical emergency and resuscitation (VMER), or other transportation means. We also included in this category the hospital admission service (emergency department, cardiac intensive/intermediate care unit, hemodynamic laboratory, cardiology nursing or other).

#### 2.2.4 Laboratory parameters

The laboratory parameters included haemoglobin, platelet count, glucose, HbA1c, total cholesterol, HDL cholesterol, LDL cholesterol, triglycerides, creatinine, troponine, brain-type natriuretic peptide (BNP)/N-terminal pro-brain natriuretic peptide (NT-proBNP), biomarkers of myocardial injury, lipid profile, and apolipoproteins A and B.

#### 2.2.5 Diagnostic procedures

The non-invasive diagnostic procedures considered were 12-lead electrocardiogram, Doppler echocardiogram, exercise stress test, myocardial perfusion scintigraphy (under physical/pharmacological stress and at rest), cardiac computed tomography angiography, and cardiac magnetic resonance imaging (including both morphological and functional assessments).

#### 2.2.6 Coronary angiography

The procedure chosen was left heart catheterisation with selective coronary angiography.

#### 2.2.7 Reperfusion strategy

Reperfusion techniques included in this category were pharmacological (fibrinolysis) or mechanical (primary angioplasty).

#### 2.2.8 Coronary angioplasty

Coronary angioplasty category encompasses four main components: the procedure itself, the medical devices used (drug-eluting stent, bare metal stent, balloon catheter or other), the operating room components and the additional hospitalization days for recovery (6 days). The coronary angioplasty was determined based on the arteries intervened: left main, left anterior descending, circumflex, right coronary artery, as well as the bypass as an additional procedure.

#### 2.2.9 CABG

The intervention considered was myocardial revascularisation, which included the operating room, blood products, inpatient stay and consultations.

#### 2.2.10 Other interventions

The category of other interventions included numerous procedures performed during hospitalization. We considered both the procedure and the medical device for the following implementations: cardiac resynchronization therapy (CRT) device, implantable cardioverter defibrillator (ICD) device, ICD + CRT (device with simultaneous capacity of cardioverter defibrillator and cardiac resynchronization therapy), Swan-Ganz catheter (pulmonary artery catheterization), intra-aortic balloon, definitive pacemaker, temporary pacemaker and ventricular assistance. As for invasive mechanical ventilation and non-invasive mechanical ventilation, only the procedure was considered.

#### 2.2.11 Drug use

The medications included those used in common clinical practice for these conditions (complete list in Unit valuation description of Supplementary material).

#### 2.2.12 Complications

We considered each of the following complications that occurred during hospitalization: (re)-infarction, heart failure, mechanical complications, sustained ventricular tachycardia, cardiac arrest resuscitated, stroke and major haemorrhage.

#### 2.2.13 Rehabilitation

Cardiac rehabilitation intervention was considered.

#### 2.2.14 Death

Information was used about the vital status of the patient at the time of the hospital discharge.

### 2.3 Unit valuation and costing

All units were valuated accordingly to official sources, as described in “Unit valuation description,” in Supplementary Table 1.

The inpatient day category was calculated by multiplying unit values by LOS. The daily cost for each drug was determined, whose average corresponds to the average daily price to be applied to each drug group. This daily cost was multiplied, if indicated, according to each patient’s LOS. Operating room costs were included in the primary angioplasty component of the reperfusion strategy category and in the coronary angioplasty category. For the remaining categories (medical transport, laboratory parameters, diagnostic procedures, coronary angiography, reperfusion strategy, coronary angioplasty, CABG, other interventions, complications, rehabilitation, death) the costs were directly imputed. The total individual cost per patient was calculated by adding up all the expenses in each category.

### 2.4 Cost inflation adjustment

Each cost item considered for each cost category was adjusted to the inflation rate (Consumer Price Index Variation Rate) to the year 2022. To adjust all costs to the same year (2022), an adjustment factor was calculated by multiplying the respective annual inflation rate by the corresponding subsequent years up to 2022. This was done for each component based on the year of the source used.

### 2.5 Potential cost determinants/factors

The year of occurrence (2002 to 2022) provides temporal context and allows for trend analyses. Admission diagnosis (STEMI, NSTEMI, UA, and undetermined AMI) distinguishes between ACS subtypes, offering insights into varying clinical presentations (12). The sex (female or male) and age group (<55, 55–75, >75) are key demographic factors possible influencing ACS incidence and outcomes, as they determine the severity of the condition, the complexity of treatment required, and the likelihood of complications, all of which significantly impact healthcare costs (12, 18). Vital status (alive or dead) provides information on patient mortality rates and associated costs. Inpatient mortality tends to result in higher costs due to increased resource consumption, such as longer length of stay (19). Lastly, the geographic regional classification (North region, Centre region, Lisbon and Tagus Valley region, Alentejo region and Algarve region, Autonomous Region of the Azores and Autonomous Region of Madeira) accounts for potential disparities in healthcare infrastructure and resource availability. By quantifying these variations, valuable spatial insights are provided, as although Portugal has a universal access NHS, regional disparities in coverage and access may emerge (20). The presence of cardiovascular risk factors, including a prior history of diabetes mellitus, dyslipidaemia, hypertension, and smoking, as well as cardiovascular history, including angina pectoris, myocardial infarction, stroke/transient ischemic attack, and peripheral vascular disease, was also considered due to their recognised impact on ACS prognosis, often leading to prolonged hospitalization and increased treatment costs. Including these comorbid illness factors enables a more precise estimation of the economic implications of ACS (21–24).

### 2.6 Statistical analysis

Given the skewed nature of the cost data (skewness of 5.02), generalized linear models (GLM) were tested using different distributions (gamma and gaussian, with identity and log link function), with the most adequate distribution being selected based on the Akaike Information Criteria (AIC) (25). Deviance residuals were further assessed to evaluate model fit (Supplementary Table 2). The significance level was established with a *p*-value threshold of <0.05. Given the minimal proportion of zero-cost observations (0.04%), their exclusion was deemed unlikely to materially affect the results, as these observations are probably a result of missing resource data, considering patients were all hospitalised. We present the results as marginal effects, in order to get estimates that can be directly interpreted in terms of cost differences (in euros). Statistical analysis, including demographic and cost analysis, was conducted using R 4.3.3 (R Foundation for Statistical Computing, Vienna, Austria).

Therefore, a regression analysis was performed to investigate the factors related to individual total costs. First, a first GLM was fitted, assuming a gamma distribution, based on goodness-of-fit results, incorporating age as a continuous variable alongside the categorical variables of year, sex, diagnosis upon admission, geographic region, and vital status upon hospital discharge. A second model was tested, incorporating the same variables of the first model but considering year as a continuous variable. A third model was tested, maintaining the assumptions of the first model while incorporating risk factors variables (cardiovascular risk factors and cardiovascular history).

### 2.7 Sensitivity analyses

To account for uncertainty and its impact on the overall mean cost, a univariate sensitivity analysis was conducted, whereby the cost of each resource was modified by an arbitrary increase or a decrease of 20%. The mean total cost was therefore recalculated.

#### 2.7.1 Decrease simulation

For each selected resource cost, 20% of the non-zero observations were randomly selected and set to zero. The non-zero observations were selected for modification to reflect a reduction in the frequency (and costs) of each procedure. This scenario thus simulates the removal of 20% of the procedures (and costs) associated with each variable.

#### 2.7.2 Increase simulation

For each selected resource cost, 20% of zero-value observations were randomly selected and set to the average cost of the non-zero observations for that variable. The zero values were selected for modification to represent an increase in the frequency (and costs) of each procedure. This scenario thus simulates the increase of the number of procedures (and costs) associated with that variable.

### 2.8 Total economic burden of ACS for the Portuguese NHS

To assess the annual total number of admissions at Portuguese NHS hospitals with ACS as the primary diagnosis, the In-patient Morbidity Database for the available years 2002 to 2018 was used, including administrative data on all hospitalizations at the Portuguese NHS. We filtered ICD-9 codes 410 and 411.1 and ICD-10 codes I21 and I20, for AMI and UA, respectively. To calculate the overall number of cases from 2019 to 2022, we determined the yearly fluctuations in the number of cases within the ProACS during those years. These variations were then used to predict the number of cases for the following years from the last year available in the In-Morbidity Database (2018).

The total annual cost for the ACS hospitalizations in the NHS was calculated by adding the total AMI and UA annual cost. The total AMI and UA annual cost were calculated by multiplying the average annual AMI cost (by averaging STEMI, NSTEMI and undetermined AMI costs) and UA cost by the total number of AMI and UA hospitalizations in the NHS. The total direct economic burden of ACS on the NHS corresponds to the sum of all the total annual costs. Furthermore, the percentage burden of the total cost of ACS on the current health expenditure of the Portuguese NHS by year was calculated by dividing each total annual ACS cost by the annual current health expenditure (current prices) on the Portuguese NHS (26). All formulas are presented in Supplementary material.

## 3 Results

### 3.1 Patient characteristics

**Figure 1:**
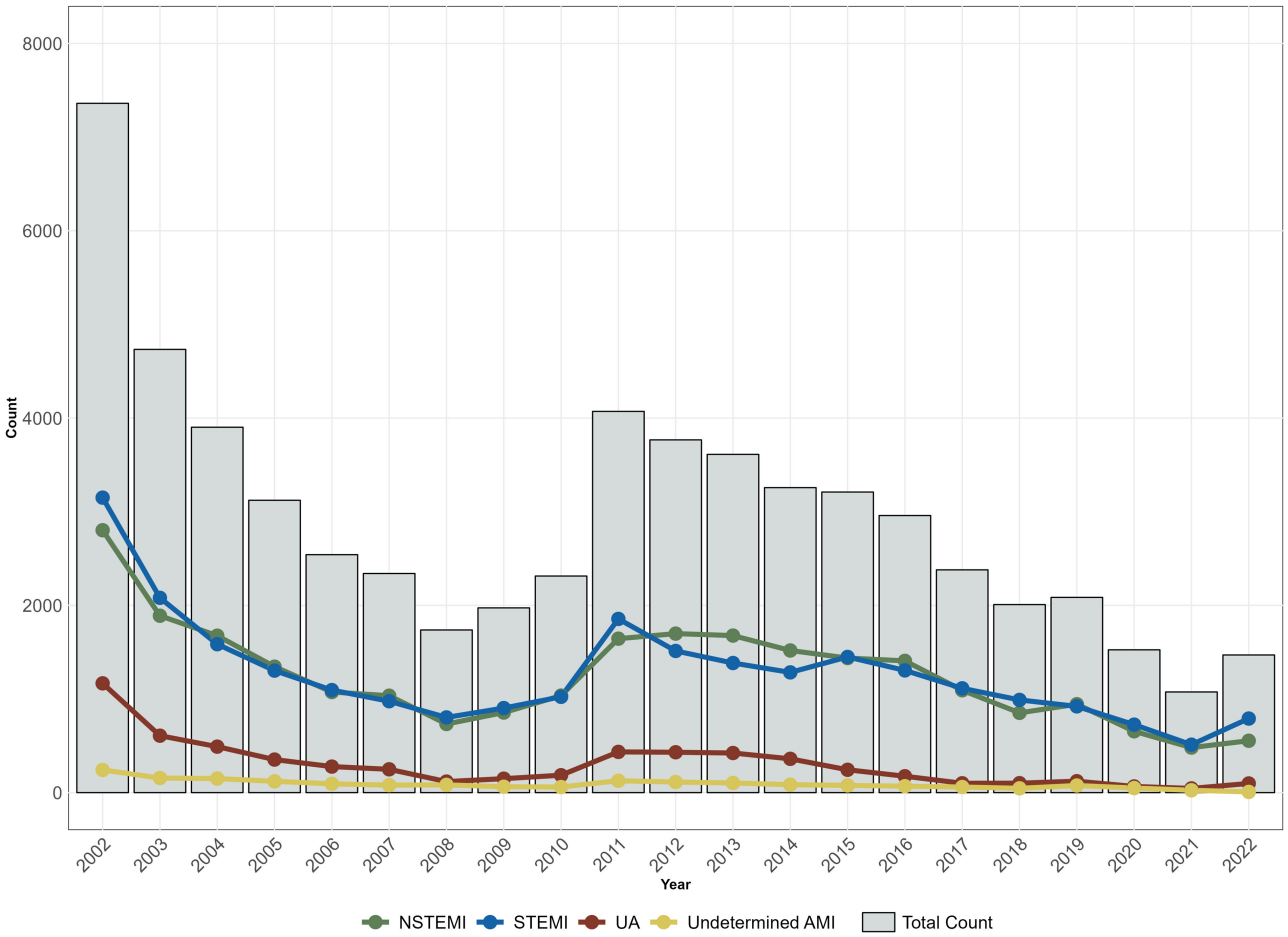
Evolution of the annual number of cases, total and by diagnosis at admission in ProACS.

Analysing the distribution of ACS cases over the 20-year period, STEMI shows a steady increase, peaking at 54.51% in 2022. NSTEMI exhibits variability, declining to 38.18% by 2022. UA cases diminish from 15.85 to 6.75%, while undetermined AMI cases remain consistently low at 0.55% in 2022 (Figure 1).

**Figure 2:**
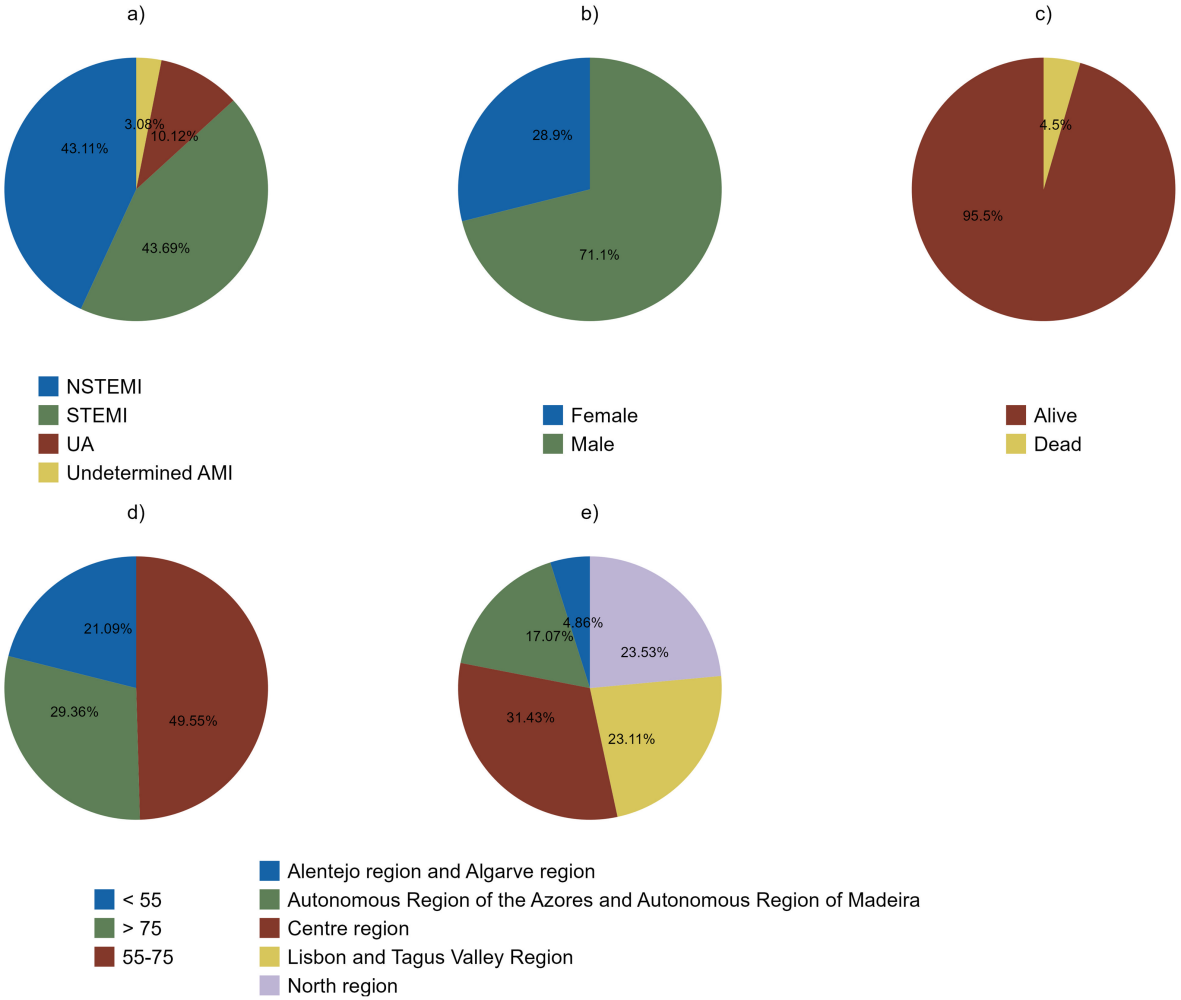
Descriptive analysis of ProACS’ proportion of cases by diagnosis at admission **(a)**, sex **(b)**, vital status upon hospital discharge **(c)**, age **(d)**, and regions **(e)** from 2002 to 2022.

Of all cases assessed, as presented in Figure 2, STEMI and NSTEMI proportions were similar at 43.69 and 43.11%, respectively. UA represented 10.12% of cases and undetermined AMI 3.08%. Also, 71.10% were male, and 95.50% survived their episode. Most cases occurred in the 55–74 age range (49.55%), and in the Centre region (31.43%). The annual relative frequencies of ProACS patient characteristics are presented in Supplementary Table 3.

### 3.2 Average costs

The interventional cardiology procedure categories were the most expensive, with CABG incurring an average cost of 6,684.68€ and coronary angioplasty of 3,198.44 € (Table 1). The category of “other interventions” also exhibited a high average cost, of 18,695.59 €.

**Table 1:** Average cost categories statistics between 2002 and 2022.

Cost category	Mean (€)	Median (€)	SD (€)	Q1 (€)	Q3 (€)	Minimum (€)	Maximum (€)	Number of observations
Daily inpatient	1,068.21	731.19	1,266.88	584.95	1,169.90	146.24	50,744.59	60,289
Medical transport	82.86	83.52	21.98	66.66	89.11	49.80	116.24	23,604
Laboratory parameters	55.32	7.49	92.19	2.46	60.74	1.33	277.76	372,027
Diagnostic procedures	70.25	67.92	25.92	67.92	67.92	32.93	366.20	28,503
Coronary angiography	546.26	546.26	0.00	546.26	546.26	546.26	546.26	47,683
Reperfusion strategy	1,927.50	2,759.78	1,236.28	91.20	2,759.78	91.20	2,759.78	17,715
Coronary angioplasty	3,198.44	3,514.81	799.88	3,313.47	3,514.81	377.03	3,514.81	37,969
CABG	6,684.68	6,684.68	0.00	6,684.68	6,684.68	6,684.68	6,684.68	558
Other Interventions	18,695.59	3,148.01	18,609.95	2,177.19	38,667.46	610.52	108,138.39	2,859
Pharmacological treatment	141.62	53.15	320.36	19.10	113.92	0.09	15,377.18	56,397
Complications	2,684.95	2,923.14	1,106.25	2,923.14	2,923.14	117.06	6,150.62	14,492
Cardiac Rehabilitation	32.73	32.73	0.00	32.73	32.73	32.73	32.73	5,604
Death	4,001.64	4,001.64	0.00	4,001.64	4,001.64	4,001.64	4,001.64	2,763

Overall, the average cost per ACS patient from 2002 to 2022 was 6,280.79 €. In terms of the average cost per diagnosis over the 21 years, STEMI had the highest value, at 7,973.00 €, while values for NSTEMI 5,203.33 €, for UA 3,464.42 € and for undetermined AMI 6,932.59 (Table 2).

**Table 2:** Average individual cost statistics per patient by overall, admission diagnosis, sex, vital status upon hospital discharge and age group.

	Mean (€)	Median (€)	SD (€)	Q1 (€)	Q3 (€)	Minimum (€)	Maximum (€)
Overall							
	6,280.79	5,072.26	7,588.87	1,874.83	8,077.81	0.92	167,185.70
Year							
2002	5,601.78	3,822.43	8,555.82	1,545.40	6,195.07	0.92	67,016.57
2003	4,880.95	3,784.12	6,527.54	1,557.35	5,781.24	11.22	58,208.83
2004	5,010.55	4,289.57	6,380.53	1,678.43	5,899.69	0.92	68,415.32
2005	5,847.10	4,837.81	7,126.73	1,796.24	7,486.96	3.20	67,424.79
2006	6,301.37	5,006.23	7,615.57	2,005.79	7,795.74	155.54	60,090.02
2007	6,251.42	5,007.66	7,145.48	2,225.82	7,774.69	155.56	61,414.81
2008	6,719.08	5,226.04	6,908.75	2,587.56	8,097.29	277.76	59,528.74
2009	7,181.78	5,403.38	7,909.47	3,551.06	8,124.69	155.34	56,950.43
2010	6,899.37	5,474.31	7,347.30	2,297.59	8,339.53	146.24	68,934.18
2011	7,745.06	5,741.50	8,998.04	3,443.82	8,746.71	211.75	167,185.68
2012	7,250.35	5,636.04	7,884.37	2,577.29	8,556.24	143.83	75,958.54
2013	6,673.40	5,481.84	6,671.28	2,310.90	8,433.06	143.83	60,319.63
2014	6,913.34	5,509.05	7,776.47	2,170.68	8,518.14	89.11	162,793.56
2015	6,818.75	5,577.13	6,712.10	2,454.42	8,520.78	49.80	64,306.15
2016	6,926.74	5,559.48	7,219.44	2,387.30	8,519.10	211.75	88,284.94
2017	6,870.36	5,573.93	7,538.59	2,025.51	8,492.67	49.80	64,693.89
2018	6,364.31	5,251.58	7,514.30	1,841.43	8,184.62	49.80	71,614.10
2019	5,736.19	4,938.38	6,884.27	1,395.17	8,139.37	83.52	67,056.71
2020	7,198.93	5,459.88	8,526.03	1,833.88	8,622.08	86.46	66,303.31
2021	4,990.73	3,023.87	5,572.58	1,507.03	7,907.08	72.74	55,554.06
2022	4,105.73	1,848.02	9,087.54	1,109.34	5,079.70	40.01	166,129.38
Admission diagnosis							
STEMI	7,973.00	7,562.40	8,942.67	2,785.83	8,695.32	0.92	167,185.68
NSTEMI	5,203.33	4,709.79	6,062.17	1,797.28	5,866.67	40.01	71,053.33
UA	3,464.42	1,974.12	4,175.63	1,092.46	4,888.06	0.92	58,208.83
Undetermined AMI	6,932.59	4,942.72	9,177.47	2,094.89	7,714.44	277.76	63,731.35
Sex							
Female	6,246.24	4,876.30	8,007.74	1,787.82	8,017.10	0.92	166,129.38
Male	6,299.53	5,143.80	7,415.77	1,914.03	8,095.76	0.92	167,185.68
Vital status							
Alive	5,727.56	4,971.37	6,195.08	1,813.55	7,945.44	0.92	121,269.00
Dead	18,029.57	10,005.83	17,921.05	7,515.75	17,623.14	4,005.76	167,185.70
Age group							
<55	6,118.24	5,179.74	7,090.07	1,844.66	8,030.50	12.14	158,810.24
55–75	6,319.50	5,103.04	7,759.56	1,910.55	8,087.95	0.92	167,185.68
>75	6,335.53	4,887.65	7,615.24	1,850.87	8,109.11	3.20	120,345.44

**Figure 3:**
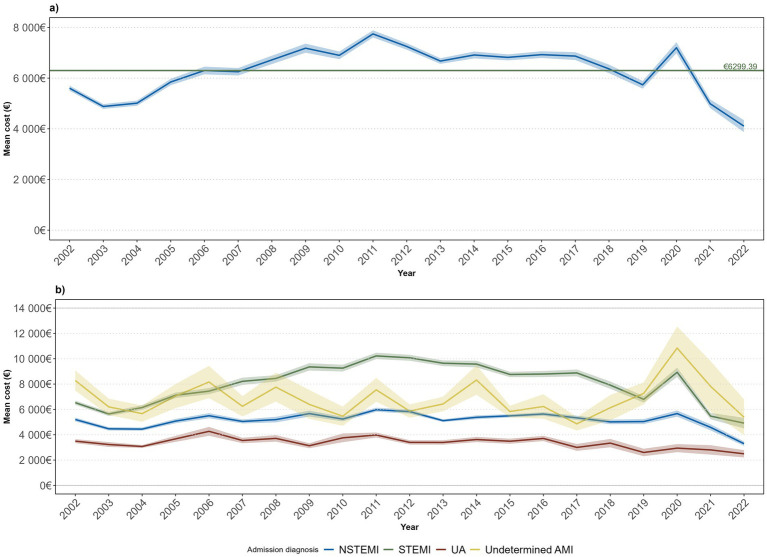
Average annual individual costs evolution (2002–2022) by ACS case **(a)** and admission diagnosis **(b)**.

An upward trend in costs was noted from 2003 to 2011. Over the following years, the average cost gradually decreased up to 2022, apart from 2020. According to the trends in the average cost per admission diagnosis, STEMI had the highest average cost over the longest period, followed by undetermined AMI, with an unstable trend, and then NSTEMI and UA, with a steadier pattern (Figure 3).

**Figure 4:**
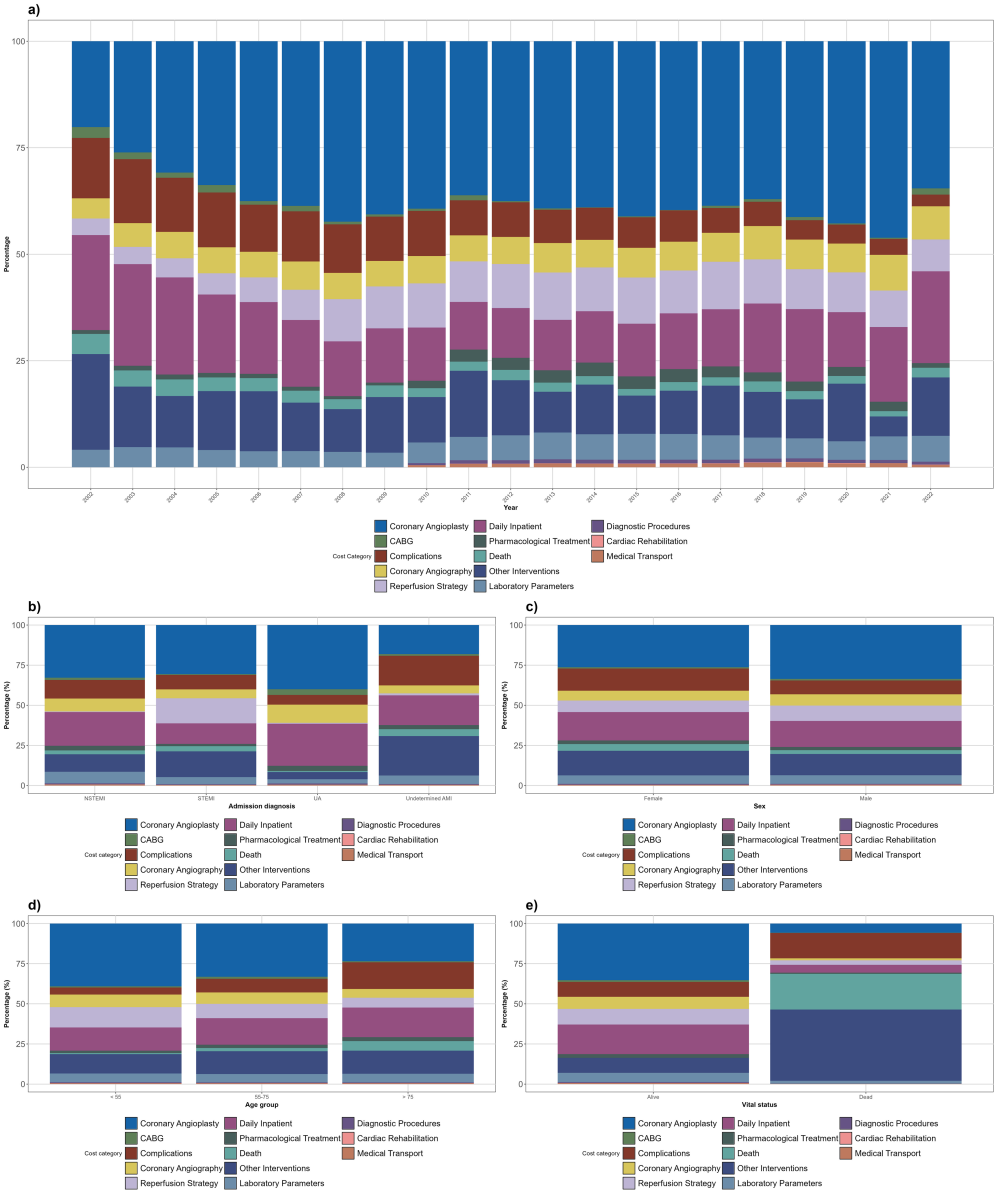
Distribution of average annual costs by resource categories, and its evolution between 2002 and 2022 (in percentage) by year **(a)**, admission diagnosis **(b)**, sex **(c)**, age group **(d)**, and vital status upon hospital discharge **(e)**.

Over time, coronary angioplasty was the dominant cost factor. The daily inpatient followed as the second highest cost factor and, despite decreasing from 2002 to 2011, registered a consecutive growth in subsequent years (excluding 2020). The other interventions also accounted for a relevant proportion of costs, regardless of having an inconsistent evolution. Additionally, coronary angiography presented a slightly more consistent growth, while the complications decreased gradually. To a lesser extent, there were minor annual fluctuations in laboratory parameters and pharmacological treatment. The remaining categories accounted for less than 5% (Figure 4).

### 3.3 Regression analysis

The selected distribution was Gamma with a log link function. STEMI patients were associated with a statistically significant excess cost of 3,853.26 € (*p* < 0.001) when compared to UA (Table 3). The same for NSTEMI and undetermined AMI, with a higher cost of 1,308.91 € (*p* < 0.001) and 2,611.70 € (*p* < 0.001), respectively. Dying is associated with a statistically significant higher value of 12,017.64 € (*p* < 0.001), compared to survivors. In contrast, each additional year in age is linked to a lower value by 0.72 € (although not statistically significant) in the total individual cost. Regarding sex, males demonstrate an excess cost on the total individual cost of 101.65 € compared to females. A statistically significant and positive association was found for the years 2005 to 2022 (*p* < 0.05) compared to 2002, indicating substantial average increases in the total individual cost, ranging from 393.25 € to 2,464.65 €. Considering the comorbidities (model 3), diabetes mellitus, smoking, and peripheral vascular disease significantly increased costs, while a history of myocardial infarction was associated with reduced costs. The findings from models 1 and 2 yielded similar results, with the male sex excess cost becoming statistically significant (*p* < 0.05) by 123.52 € and 154.52 €, respectively. Furthermore, when the year was considered as a continuous variable (model 2), costs increased by 72.80€ (*p* < 0.001) for each additional year (Table 3).

**Table 3:** Results of multivariate regression: adjusted marginal effects.

Predictors	Model 1 (AME, CI)		Model 2 (AME, CI)		Model 3 (AME, CI)	
Sex						
Male	123.52*	[2.52, 244.51]	154.52*	[34.71, 274.34]	101.65	[−27.85, 231.14]
Female [REF]						
Admission diagnosis						
STEMI	3,761.46***	[3,615.31, 3,907.61]	3,819.71***	[3,675.77, 3,963.66]	3,853.26***	[3,690.87, 4,015.65]
NSTEMI	1,367.95***	[1,241.01, 1,494.88]	1,401.73***	[1,276.98, 1,526.49]	1,308.91***	[1,173.52, 1,444.30]
Undetermined AMI	2,778.77***	[2,435.46, 3,122.08]	2,792.86***	[2,454.07, 3,131.65]	2,611.7***	[2,268.47, 2,954.93]
UA [REF]						
Regions						
North region	1,360.25***	[1,208.85, 1,511.65]	1,137.45***	[987.50, 1,287.40]	996.66***	[839.77, 1,153.55]
Lisbon and Tagus Valley region	1,215.61***	[1,066.15, 1,365.06]	727.2***	[585.74, 868.67]	1108.65***	[947.50, 1,269.81]
Alentejo region and Algarve region	503.88***	[255.37, 752.38]	284.98*	[39.31, 530.65]	259.47*	[8.57, 510.37]
Autonomous region of the Azores and Autonomous region of Madeira	1,223.47***	[1,060.55, 1,386.38]	1,116.17***	[953.76, 1,278.57]	871.93***	[702.66, 1,041.20]
Centre region [REF]						
Vital status						
Dead	11,525.42***	[10,811.78, 12,239.06]	11,344.53***	[10,643.79, 12,045.27]	12,017.64***	[11,232.21, 12,803.08]
Alive [REF]						
Age						
	−2.25	[−6.61, 2.12]	−2.61	[−6.94, 1.73]	−0.72	[−5.94, 4.50]
Year (discrete)						
	-	-	72.8***	[63.49, 82.10]	-	-
Year (continuous)						
2003	−464.45***	[−665.58, -263.32]	-	-	−468.7***	[−667.27, -270.13]
2004	−270.67*	[−492.93, -48.41]	-	-	−266.67*	[−486.37, -46.96]
2005	407.6**	[146.39, 668.80]	-	-	393.25**	[135.73, 650.76]
2006	868.79***	[576.31, 1,161.27]	-	-	876.2***	[587.13, 1,165.27]
2007	692.97***	[402.35, 983.58]	-	-	756.65***	[466.96, 1,046.33]
2008	1,168.62***	[817.92, 1,519.32]	-	-	1,234.98***	[885.67, 1,584.28]
2009	1,542.42***	[1,180.54, 1,904.31]	-	-	1,662.78***	[1,297.55, 2,028.01]
2010	1,514.26***	[1,185.90, 1,842.62]	-	-	1,609.74***	[1,261.76, 1,957.71]
2011	2,568.98***	[2,268.22, 2,869.74]	-	-	2,464.65***	[2,149.37, 2,779.92]
2012	2,132.01***	[1,843.79, 2,420.22]	-	-	2,163.23***	[1,862.70, 2,463.75]
2013	1,693.65***	[1,413.96, 1,973.35]	-	-	1,657.46***	[1,366.14, 1,948.78]
2014	1,949.88***	[1,650.41, 2,249.35]	-	-	1,925.11***	[1,612.72, 2,237.50]
2015	1,816.02***	[1,520.59, 2,111.44]	-	-	1,885.55***	[1,574.44, 2,196.66]
2016	1,782.54***	[1,478.41, 2,086.66]	-	-	1,858.39***	[1,535.93, 2,180.85]
2017	1,358.65***	[1,041.06, 1,676.24]	-	-	1,623.77***	[1,279.10, 1,968.43]
2018	946.29***	[620.66, 1,271.92]	-	-	1,151.36***	[806.97, 1,495.75]
2019	533.28***	[230.18, 836.38]	-	-	931.81***	[596.94, 1,266.67]
2020	1,664.48***	[1,261.89, 2,067.07]	-	-	2,460.59***	[1,984.13, 2,937.06]
2021	−259.69	[−610.84, 91.46]	-	-	859.88***	[371.92, 1,347.84]
2022	−1,663.77***	[−1,906.96, −1,420.58]	-	-	723.5*	[154.91, 1,292.10]
2002 [REF]						
Diabetes mellitus						
Yes	-	-	-	-	696.21***	[561.10, 831.32]
No [REF]						
Dyslipidaemia						
Yes	-	-	-	-	2.15	[−117.78, 122.08]
No [REF]						
Hypertension						
Yes	-	-	-	-	−32.45	[−162.28, 97.38]
No [REF]						
Smoking						
Yes	-	-	-	-	272.94***	[118.15, 427.72]
No [REF]						
Angina pectoris						
Yes	-	-	-	-	88.66	[−50.19, 227.51]
No [REF]						
Myocardial infarction						
Yes	-	-	-	-	−148.75*	[−296.77, −0.72]
No [REF]						
Stroke/transient ischemic attack						
Yes	-	-	-	-	118.99	[−110.11, 348.08]
No [REF]						
Peripheral vascular disease						
Yes	-	-	-	-	374.46*	[72.14, 676.77]
No [REF]						

Significance levels are indicated by * for *p* < 0.05, ** for *p* < 0.01 and *** for *p* < 0.001. Average marginal effects (AME). Confidence interval (CI).

### 3.4 Sensitivity analyses

The analysis revealed that while most variables demonstrated minimal deviation in the average cost per patient when the parameter was modified, certain key resources variations provoked notable average cost variations. Specifically, there was an increase of about 10% for coronary angioplasty and almost 21% for CABG. For complications costs, the average cost increased by up to 19.53% (for major bleeding complications costs). For mortality costs, there was an average cost increase of 12%. Nevertheless, the largest increases were observed among the other interventions costs, particularly for the CRT, DCI + CRT and DCI variables (62.44, 62.43 and 46.71%, respectively), for invasive mechanical ventilation (120.62%) and for ventricular assist device (344.29%). All resources are detailed in the Supplementary Table 6.

### 3.5 Direct economic burden

Considering a total of 253,269 hospitalizations for AMI and 41,038 hospitalizations for UA, the total direct economic burden of ACS on the Portuguese NHS over the 21 years was estimated at 1,831,280,867.38 € over the period. From 2002 to 2004, there was a gradual decline in total annual expenditure, followed by an intermittent upward trend in the next decade, peaking in 2014 at 106,190,390.80 €. Thereafter, expenditure gradually declined until 2021, reaching a lowest of 44,348,962.21 €, with a minor rise the following year. The percentage burden of ACS on the total health expenditure of the Portuguese NHS peaked in 2002 at 1.21% (Table 4).

**Table 4:** Total economic burden of ACS for the Portuguese NHS.

	ICD-9 code 410/ ICD-10 code I21	ICD-9 code 4111/ICD-10 code I20	Average AMI (STEMI, NSTEMI and Undetermined AMI) cost	Average UA cost	Total annual AMI cost (€)	Total annual UA cost (€)	Total annual ACS cost (€)	Annual current health expenditure (current prices) on the Portuguese National and Reginal Health Service (€)	Annual percentage burden of the total cost of ACS on the current health expenditure of the Portuguese National and Regional Health Service (%)
2002	11,440	2,958	6,672.01	3,494.71	76,327,840.16	10,337,361.05	86,665,201.21	7,147,814,000.00	1.21
2003	12,397	2,475	5,436.29	3,228.12	67,393,699.53	7,989,606.90	75,383,306.43	7,536,536,000.00	1.00
2004	12,315	2,225	5,419.61	3,073.45	66,742,435.58	6,838,419.58	73,580,855.15	8,161,580,000.00	0.90
2005	11,836	1,850	6,415.66	3,675.16	75,935,751.76	6,799,042.30	82,734,794.06	8,661,447,000.00	0.96
2006	11,967	1,647	7,035.93	4,270.12	84,198,986.28	7,032,887.64	91,231,873.92	8,544,438,000.00	1.07
2007	12,467	1,768	6,503.82	3,546.68	81,083,086.54	6,270,521.40	87,353,607.94	8,753,442,000.00	1.00
2008	12,823	1,744	7,133.29	3,713.11	91,470,126.38	6,475,663.84	97,945,790.22	9,200,945,000.00	1.06
2009	12,456	1,396	7,140.44	3,133.68	88,941,270.82	4,374,620.07	93,315,890.89	9,773,703,000.00	0.95
2010	12,467	1,189	6,652.03	3,756.81	82,930,795.68	4,466,848.28	87,397,643.95	10,562,753,000.00	0.83
2011	12,400	1,169	7,915.12	3,979.29	98,147,537.60	4,651,794.69	102,799,332.29	9,647,164,000.00	1.07
2012	12,683	1,327	7,256.47	3,397.83	92,033,770.96	4,508,923.06	96,542,694.03	8,947,239,000.00	1.08
2013	12,832	1,550	7,061.42	3,393.05	90,612,179.94	5,259,225.95	95,871,405.89	8,967,773,000.00	1.07
2014	12,950	1,581	7,757.75	3,622.77	100,462,797.75	5,727,593.05	106,190,390.80	8,992,842,000.00	1.18
2015	13,256	1,663	6,694.25	3,487.54	88,738,938.23	5,799,779.02	94,538,717.25	9,131,257,000.00	1.04
2016	13,390	2,439	6,885.97	3,707.37	92,203,165.08	9,042,280.31	101,245,445.39	9,521,809,000.00	1.06
2017	14,144 ^p^	3,276 ^p^	6,358.72	2,999.63	89,937,721.54	9,826,797.71	99,764,519.24	9,849,083,000.00	1.01
2018	12,655 ^p^	2,652 ^p^	6,355.30	3,339.80	80,426,296.19	8,857,138.99	89,283,435.18	10,405,443,000.00	0.86
2019	13,140 ^e^	2,754 ^e^	6,365.72	2,600.75	83,647,359.67	7,161,670.09	90,809,029.76	10,927,175,000.00	0.83
2020	9,611 ^e^	2,014 ^e^	8,482.65	2,943.66	81,526,703.84	5,928,798.79	87,455,502.63	11,878,928,000.00	0.74
2021	6,775 ^e^	1,420 ^e^	5,958.49	2,803.57	40,368,533.65	3,980,428.57	44,348,962.21	13,225,444,000.00 ^Po^	0.34
2022	9,264 ^e^	1,941 ^e^	4,530.98	2,495.98	41,976,636.20	4,845,832.75	46,822,468.95	14,241,594,000.00 ^Pe^	0.33
Total	253,269	41,038	-	-	1,695,105,633.35	136,175,234.03	1,831,280,867.38	-	-

p Provisional.

e Estimated.

Po Provisional value.

Pe Preliminary value.

## 4 Discussion

### 4.1 Key findings

ACS accounts as one of the primary causes of morbidity and mortality in developed nations, including Portugal. One direct implication is the significant financial burden on healthcare systems, which in Portugal is primarily borne by the NHS. As the first investigation to examine this disease and employ a bottom-up approach in direct cost analysis, between 2002 and 2022 the average individual cost per ACS episode in Portugal was 6,280.79 €. Our study revealed notable variations in average costs among different admission diagnosis, sex groups, vital status and age groups. For instance, STEMI and NSTEMI patients had an increased cost of 3,853.26 € and 2,611.70 €, respectively, compared to UA, while deceased patients had an increased cost of 12,017.64 € compared to alive patients.

Over the 21 years, the economic burden of ACS on the Portuguese NHS was 1,831,280,867.38 €, with an average annual total cost of 87,203,850.83 €. On average, this accounted for 0.93% of the NHS’s annual health expenditure.

### 4.2 Comparison with international evidence

At the European level, the costs presented for the Portuguese context are consistent with the cost patterns already reported for other countries, although lower. In Spain, a study from the public perspective reported an ACS average individual direct cost of 12,252.9 €, with STEMI costing 12,245.7 € and NSTEMI 12,264.7 €. It should be noted that their follow-up lasted 2 years, which may justify the higher costs compared to our study, had a follow-up of one-year (8). As for a selected area part of the Italian NHS, the estimated average annual direct cost per patient was 11,464 €, 12,112 € for men and 10,307 € for women, with lower costs in the oldest age group compared to the younger groups (9). An analogous Italian study from a different area reported similar outcomes, with STEMI costing more than NSTEMI and UA, respectively. Additionally, female, older and surviving patients demonstrated lower average costs, as demonstrated by our study (10). In a Swedish study, the reported cost per AMI during the first year of follow-up was 12,460 €, with higher costs in younger patients (11). Once again, these results are in accordance with ours. The slightly lower costs estimated for Portugal reflect Portugal’s economic status, a lower-income nation compared to the above-mentioned and, marked by lower salaries and cheaper medical devices and drugs.

Focusing on the total direct annual costs, which average over eighty-seven million euros (as demonstrated by our research), it becomes evident that the magnitude of the estimation is substantial when compared to the previously estimated overall indirect cost burden of over 10 million (14) (approximately nine times higher).

### 4.3 Interpreting temporal trends

Considering the extensive timeframe under review, it is necessary to account for changes in clinical guidelines, as these inevitably lead to fluctuations in the average individual cost, thus allowing for the definition of relevant key periods (27).

From 2002 to 2011, there was an upward trend in costs, possible attributable to the increase in revascularization procedures (in detriment of fibrinolysis), which resulted in higher costs. However, the European Society of Cardiology’s recommendations (relevant to the Portuguese context) changed between 2011 and 2019, encouraging physicians to adopt a strategy culprit lesion only and incomplete revascularization at the index hospitalization, leaving other lesions to be treated latter on if necessary. As a result, the average annual cost decreased, which represented the downward trend. Another notable alteration was the shift from the surgical strategy of treating ACS with CABG, which is currently very residual, to coronary angioplasty, which from the 2010s became the prevailing treatment alternative (Table 3). Considering that the unitary procedure cost of coronary angioplasty is about half the CABG (Supplementary Table 1), a reduction in average annual costs is justifiable.

We must also point out that in 2020 there was an increase in the average cost possibly because of specific conditions related to the COVID-19 pandemic. Along with an overall reduction in ACS-related hospitalizations, also evident in ProACS, patients were more reluctant to go to hospital, with substantial delays and, when admitted, could display more severe presentations (28). Specifically, the major contributor to the increase in costs was the growth in the share of the other interventions cost category (Supplementary Table 4), particularly and interestingly the need for invasive mechanical ventilation (Supplementary Table 5), which entails very high expenses and was particularly demanded during the COVID-19 pandemic management. This association has been already stated (28).

In the following years, 2021 and 2022, the downward cost trend resumed, which may reflect the ability of the Portuguese NHS to maintain normal operations despite the COVID-19 pandemic.

### 4.4 Interpreting other cost drivers

STEMI, male and younger ACS cases carried a higher treatment cost (11, 12). The cost disparity between sexes, although not significant, may be attributed to a larger proportion of interventional cardiology procedures, with men accounting for 51.13% compared to women’s 40.41%. This difference is particularly evident in the cost categories of coronary angiography and coronary angioplasty. This confirms previous studies (10). These results may also reflect possible sex disparities in healthcare access and care, given that women are more likely to be misdiagnosed and undertreated (29, 30). In terms of cost differences between age groups, although also not significant, this may be due to the tendency for younger patients to be treated with more invasive and therefore more expensive treatments, as opposed to a more conservative strategy for the older population, which has been reported previously and is consistent with our findings (9, 11). As previously reported, STEMI patients had a significant higher average cost than NSTEMI patients. However, it has also been noted that NSTEMI patients are the highest resources consumers. This is supported by our findings, as NSTEMI patients had a longer LOS (Supplementary Figure 2) and a higher proportion in the most expensive categories. The sensitivity analysis findings suggest that variations in the parameters of specific resources may substantially modify the overall mean costs. Notably, while an increase in the number of interventional cardiology procedures and complications led to considerable cost increases, the most dramatic effects were observed for interventions related to the utilisation of CRT and DCI + CRT devices, invasive mechanical ventilation and also ventricular assistance. This emphasises the critical need to carefully integrate these high-cost interventions in clinical practice, closely following international guidelines (31).

### 4.5 Limitations

The ProACS is a detailed database, although presenting some limitations related with the timeframe and number of hospitals considered. There is still a possibility that some records may have omissions in the variables considered in the cost categories (e.g., not all medications taken by the patient during the hospitalization were recorded). Therefore, the total cost estimated could be lower than real cost.

Additional limitations included inconsistency in the number of participating centres over time, which limit, to some extent, the comparability of costs across years. That is, the participant hospitals may have different efficiency levels, which reflect in changes in average costs. Note, however, that a relatively large sample of hospitals was included, which may limit this bias.

In terms of whether comparable findings on changes in costs over time have been reported in studies conducted in other countries, no direct references could be identified, given the comprehensive temporal scope of our study, which differs from the more limited time horizons adopted in other studies. This distinctive feature contributes to the uniqueness of our study and addresses a gap in the existing literature.

Also, our data missed information on out-of-hospital events, e.g., consultations or interventions in other primary care settings or diagnosis facilities. Given that the main cost drivers are the intensive high-technology interventions, the cost under-estimation is not likely to be relevant. We also missed detailed information on readmissions (particularly for complications), whose value was based on prices, which are likely to be lower than real costs (considering the under-financing of NHS hospitals).

Moreover, there is no information on the number of ACS admissions in private healthcare organisations, so this analysis is only representative of the public healthcare sector.

## 5 Conclusion

Given the estimated total economic burden of ACS on the Portuguese NHS over the 21-year period considered (2002 to 2022) of 1,831,280,867.38 €, of which 93% was attributable to AMI, the cost of treating ACS is considerable and could potentially be minimised. Hence, prioritized prevention and implement programs aiming to reduce exposure to the risk factors of the disease (e.g., environmental, behaviours, diet) is fully justified (32).

A number of prevention and treatment interventions have been identified as cost-effective for CVD in both a population (or health system) and individual context. Effective (environmental) interventions at the population level include the implementation of taxation and regulation to influence dietary behaviour, including an increase in healthy food and a reduction in salt and trans fatty acids, as well as physical activity interventions (33–37). Tobacco taxation and smoking cessation programs have also demonstrated cost-effectiveness (34–36, 38, 39). In the context of the health system, task sharing with community health workers and the role of primary healthcare centres has also been demonstrated to be an effective strategy (35, 36). At the individual level, the use of simplified risk control screening/diagnostic, management, treatment, and rehabilitation regimens has been proven cost-effective (34–36, 39–41). Indeed, adherence to selected drug therapies, outpatient controls and examinations, and outpatient cardiac rehabilitation programs resulted in clinical benefits for ACS patients, while only a cost saving was observed for the drug therapies. Furthermore, a superior cost-effectiveness profile emerged for AMI patients (in comparison to UA) and for patients with more severe clinical complexity (in comparison to those with milder conditions) (40).

Furthermore, investment in cost containment policies to allow the NHS to effectively control the total expenditure per ACS patient is also recommended.

## Data Availability

The datasets presented in this article are not readily available because the data underlying this article were provided by the National Center for Data Collection in Cardiology, Portuguese Society of Cardiology, Coimbra, Portugal by permission. Requests to access the datasets should be directed to National Center for Data Collection in Cardiology, Portuguese Society of Cardiology, Coimbra, Portugal.

## References

[1] INE. (2023). Óbitos (N.o) por Local de residência (NUTS-2013), Sexo, Grupo etário e Causa de morte (Lista sucinta europeia); Anual. Lisboa: Instituto Nacional de Estatística. Available online at: http://tiny.cc/dq7jvz (Accessed December 18, 2023).

[2] Eurostat. (2023). Hospital discharges by diagnosis, in-patients, per 100 000 inhabitants [hlth_co_disch2__custom_9219041]. Eurostat. Available online at: http://tiny.cc/wzssvz

[3] Eurostat (2023). Causes of death - standardised death rate by NUTS 2 region of residence [hlth_cd_asdr2__custom_9219468]. Eur Secur. Available online at: http://tiny.cc/vzssvz (Accessed January 5, 2024).

[4] Saito Y Oyama K Tsujita K Yasuda S Kobayashi Y. Treatment strategies of acute myocardial infarction: updates on revascularization, pharmacological therapy, and beyond. J Cardiol. (2023) 81:168–78. doi: 10.1016/j.jjcc.2022.07.003, PMID: 35882613

[5] INE. (2023). Índice de envelhecimento (N.o) por Sexo; Anual. Lisboa: INE. Available online at: http://tiny.cc/dmrsvz (Accessed December 18, 2023).

[6] INE. (2020). Índice de envelhecimento (projeções 2018–2080- N.o) por Local de residência (NUTS -2013) e Cenário; Anual. Lisboa: INE. Available online at: http://tiny.cc/2jqsvz (Accessed December 18, 2023).

[7] Damluji AA Forman DE Wang TY Chikwe J Kunadian V Rich MW. Management of Acute Coronary Syndrome in the older adult population: a scientific statement from the American Heart Association. Circulation. (2023) 147:e32–62. doi: 10.1161/CIR.0000000000001112, PMID: 36503287 PMC10312228

[8] Sicras-Mainar A Fernández de Bobadilla J Navarro-Artieda R Martín I Varela-Moreno C. Morbimortalidad y consumo de recursos asociados tras síndrome coronario agudo en una población española. Rev Clin Esp. (2011) 211:560–71. doi: 10.1016/j.rce.2011.07.007, PMID: 22088667

[9] Roggeri DP Roggeri A Rossi E Cinconze E De Rosa M Maggioni AP. Direct healthcare costs and resource consumption after acute coronary syndrome: a real-life analysis of an Italian subpopulation. Eur J Prev Cardiol. (2014) 21:1090–6. doi: 10.1177/2047487313483608, PMID: 23515447

[10] Roggeri A Gnavi R Dalmasso M Rusciani R Giammaria M Anselmino M. Resource consumption and healthcare costs of acute coronary syndrome: a retrospective observational administrative database analysis. Crit Pathw Cardiol. (2013) 12:204–9. doi: 10.1097/HPC.0b013e3182a78c06 24240551

[11] Janzon M Henriksson M Hasvold P Hjelm H Thuresson M Jernberg T. Long-term resource use patterns and healthcare costs after myocardial infarction in a clinical practice setting: results from a contemporary nationwide registry study. Eur Heart J Qual Care Clin Outcomes. (2016) 2:291–8. doi: 10.1093/ehjqcco/qcw019, PMID: 29474723

[12] Tran DT Welsh RC Ohinmaa A Thanh NX Kaul P. Resource use and burden of hospitalization, outpatient, physician, and drug costs in short-and long-term care after acute myocardial infarction. Can J Cardiol. (2018) 34:1298–306. doi: 10.1016/j.cjca.2018.05.022, PMID: 30170782

[13] Bishu KG Lekoubou A Kirkland E Schumann SO Schreiner A Heincelman M. Estimating the economic burden of acute myocardial infarction in the US: 12 year National Data. Am J Med Sci. (2020) 359:257–65. doi: 10.1016/j.amjms.2020.02.004, PMID: 32265010

[14] Timóteo AT Gouveia M Soares C Cruz FR. Indirect costs of myocardial infarction in Portugal. Rev Port Cardiol. (2020) 39:245–51. doi: 10.1016/j.repc.2019.09.010, PMID: 32505635

[15] Pereira JA. Revisão das orientações metodológicas de estudos de avaliação económica de medicamentos em Portugal [Revising methodological guidelines for the economic evaluation of pharmaceuticals in Portugal]. Port. J Public Health. (2019) 36:I–IV. doi: 10.1159/000495740, PMID: 40011618

[16] Timóteo AT Mimoso J. Portuguese registry of acute coronary syndromes (pro ACS): 15 years of a continuous and prospective registry. Rev Port Cardiol. (2018) 37:563–73. doi: 10.1016/j.repc.2017.07.016, PMID: 30008312

[17] Timóteo AT Mimoso J. Portuguese registry of acute coronary syndromes (pro ACS): 15 years of a continuous and prospective registry. Rev Portuguesa Cardiol. (2018) 37:563–73. doi: 10.1016/j.repce.2017.07.011, PMID: 30008312

[18] Jan S Lee SWL Sawhney JPS Ong TK Chin CT Kim HS. Predictors of high-cost hospitalization in the treatment of acute coronary syndrome in Asia: findings from EPICOR Asia. BMC Cardiovasc Disord. (2018) 18:139. doi: 10.1186/s12872-018-0859-4, PMID: 29973147 PMC6033225

[19] Page RL Ghushchyan V van den Bos J Gray TJ Hoetzer GL Bhandary D. The cost of inpatient death associated with acute coronary syndrome. Vasc Health Risk Manag. (2016) 12:13–21. doi: 10.2147/VHRM.S94026, PMID: 26893568 PMC4745827

[20] Value for Health CoLAB. (2023). Atlas da Variação em Saúde no Serviço Nacional de Saúde Português −2018. 1st ed. Value for Health CoLAB, editor.

[21] Gong W Yan Y Liu J Wang X Zheng W Que B. In-Hospital Mortality and Treatment in Patients With Acute Coronary Syndrome With and Without Standard Modifiable Cardiovascular Risk Factors: Findings From the CCC-ACS Project. J Am Heart Assoc. (2024). 13:e029252. 39291502 10.1161/JAHA.122.029252 PMC11681477

[22] Cowper PA Knight JD Davidson-Ray L Peterson ED Wang TY Mark DB. Acute and 1-Year Hospitalization Costs for Acute Myocardial Infarction Treated With Percutaneous Coronary Intervention: Results From the TRANSLATE-ACS Registry. J Am Heart Assoc. (2019). 8. 10.1161/JAHA.118.011322 PMC6507213 30975005

[23] Jan S Lee SWL Sawhney JPS Ong TK Chin CT Kim HS. Predictors of high-cost hospitalization in the treatment of acute coronary syndrome in Asia: findings from EPICOR Asia. BMC Cardiovasc Disord. (2018). 18:139. 29973147 10.1186/s12872-018-0859-4 PMC6033225

[24] Bramkamp M Radovanovic D Erne P Szucs TD. Determinants of costs and the length of stay in acute coronary syndromes: a real life analysis of more than 10,000 patients. Cardiovasc Drugs Ther. (2007). 21:389–98. 17805954 10.1007/s10557-007-6044-0

[25] Lindsey JK Jones B. Choosing among generalized linear models applied to medical data. Stat Med. (1998) 17:59–68. doi: 10.1002/(SICI)1097-0258(19980115)17:1<59::AID-SIM733>3.0.CO;2-7, PMID: 9463849

[26] INE. (2023). Despesa corrente em saúde por agente financiador (preços correntes). Lisboa: Instituto Nacional de Estatística. Available online at: http://tiny.cc/it7jvz

[27] Neumann FJ Sousa-Uva M Ahlsson A Alfonso F Banning AP Benedetto U. 2018 ESC/EACTS Guidelines on myocardial revascularization. Eur Heart J. (2019) 40:87–165. doi: 10.1093/eurheartj/ehy394, PMID: 30165437

[28] Chouairi F Pinsker B Fudim M Miller PE. Trends in outcomes and resource utilization for acute myocardial infarction admissions during the COVID-19 pandemic. Am Heart J. (2023) 258:114–8. doi: 10.1016/j.ahj.2023.01.003, PMID: 36646197 PMC9839385

[29] Zhou S Zhang Y Dong X Zhang X Ma J Li N. Sex disparities in management and outcomes among patients with acute coronary syndrome. JAMA Netw Open. (2023) 6:e2338707–7. doi: 10.1001/jamanetworkopen.2023.38707, PMID: 37862014 PMC10589815

[30] Stehli J Duffy SJ Burgess S Kuhn L Gulati M Chow C. Sex disparities in myocardial infarction: biology or Bias? Heart Lung Circ. (2021) 30:18–26. doi: 10.1016/j.hlc.2020.06.025, PMID: 32861583

[31] Weiting H Yaoxian AZ Keong YK Lam SW How LY Sahlén AO. The clinical value and cost-effectiveness of treatments for patients with coronary artery disease. Heal Econ Rev. (2022) 12:1–8. doi: 10.1186/s13561-022-00401-y, PMID: 36348165 PMC9644580

[32] Santos JV Vandenberghe D Lobo M Freitas A. Cost of cardiovascular disease prevention: towards economic evaluations in prevention programs. Ann Transl Med. (2020) 8:512–2. doi: 10.21037/atm.2020.01.20, PMID: 32395556 PMC7210201

[33] Bonekamp NE Visseren FLJ van der Schouw YT van der Meer MG Teraa M Ruigrok YM. Cost-effectiveness of Mediterranean diet and physical activity in secondary cardiovascular disease prevention: results from the UCC-SMART cohort study. Eur J Prev Cardiol. (2024) 31:1460–8. doi: 10.1093/eurjpc/zwae123, PMID: 38547043

[34] Aminde LN Takah NF Zapata-Diomedi B Veerman JL. Primary and secondary prevention interventions for cardiovascular disease in low-income and middle-income countries: a systematic review of economic evaluations. Cost Effect Resour Allocat. (2018) 16:22. doi: 10.1186/s12962-018-0108-9, PMID: 29983644 PMC6003072

[35] Gaziano T Suhrcke M Brouwer E Levin C Nikolic I Nugent R. Costs and cost-effectiveness of interventions and policies to prevent and treat cardiovascular and respiratory diseases In: Disease control priorities, third edition (volume 5): Cardiovascular, respiratory, and related disorders. Washington (DC): The World Bank (2017). 349–67. 30212069

[36] Schwalm JD McKee M Huffman MD Yusuf S. Resource effective strategies to prevent and treat cardiovascular disease. Circulation. (2016) 133:742–55. doi: 10.1161/CIRCULATIONAHA.115.008721, PMID: 26903017 PMC4766731

[37] Chokshi DA Farley TA. The cost-effectiveness of environmental approaches to disease prevention. N Engl J Med. (2012) 367:295–7. doi: 10.1056/NEJMp1206268, PMID: 22830461

[38] Leosdottir M Wärjerstam S Michelsen HÖ Schlyter M Hag E Wallert J. Improving smoking cessation after myocardial infarction by systematically implementing evidence-based treatment methods. Sci Rep. (2022) 12:642. doi: 10.1038/s41598-021-04634-5, PMID: 35022490 PMC8755785

[39] Dugani SB Moran AE Bonow RO Gaziano TA. Ischemic heart disease: cost-effective acute management and secondary prevention In: Disease control priorities, third edition (volume 5): Cardiovascular, respiratory, and related disorders. Washington (DC): The World Bank (2017). 135–55. 30212078

[40] Rea F Ronco R Martini N Pietro MA Corrao G. Cost-effectiveness of Posthospital Management of Acute Coronary Syndrome: a real-world investigation from Italy. Value Health. (2022) 25:185–93. doi: 10.1016/j.jval.2021.07.015, PMID: 35094791

[41] Callea G Tarricone R Lara AM. Economic evidence of interventions for acute myocardial infarction: a review of the literature. Euro Intervent. (2012) 8:P71–6. doi: 10.4244/EIJV8SPA12, PMID: 22917795

