# Supplementary material for: Direct economic burden of acute coronary syndromes in the Portuguese National Health Service—facts and trends between 2002 and 2022
Source: Front Public Health. 2025 Mar 18;13:1433307. doi: 10.3389/fpubh.2025.1433307 (PMC11959009; doi:10.3389/fpubh.2025.1433307)
Supplement: Supplementary file 1 [file Data_Sheet_1.PDF]

Supplementary Material

1 Supplementary Data

1.1 Unit valuation description

1.1.1 Daily inpatient

Daily inpatient costs account for hospitality costs and healthcare professional salary costs, physicians and nurses, and were derived from real-world published data and from the 2022 official salary tables for each healthcare professional, respectively. For daily hospitality, the standardized occupancy cost was calculated by the mean standardized bed occupancy cost per episode divided by the mean overall ward of hospitalisation length. For physicians, the salary amounted to one hour's worth of work for each daily round, whereas for nurses, it corresponded to an hour per eight-hour shift, equalling three hours per day[1–3].

1.1.2 Operating room

The operating room unit costs contain the fixed costs of the operating room (linen, sterilisation, external services, equipment amortisation and other costs), which correspond to 20% of the national total average cost per room hour, based on the official report of the Ministry of Health "Evaluation of the National Situation of Operating Rooms", and the salary costs of the healthcare professionals, equivalent to one hour's work for two senior haemodynamic laboratory physicians, one specialist nurse and two diagnostic and therapeutic superior technicians (one radiology technician and one cardio pneumology technician), obtained from the official salary tables 2022 for each healthcare professional. The costs of consumables, such as pharmaceuticals and medical devices, are allocated in other parcels[1,2,4,5].

1.1.3 Medical transport

Unit cost applied for medical transport were the average cost per medical transport activation of National Institute of Medical Emergency (INEM), derived for ambulances without a doctor, directly from Audit Report No. 47/2010 “Results Audit of the National Institute of Medical Emergency”, and for the Vehicle for Medical Emergency and Resuscitation (VMER), by indirect calculation using the INEM "Emergency Medical Services Activity Report 2022" and Government Orders published in Journal of the Republic. The cost of the hospital admission location was derived from Government Order n.º 207/2017, of July 11th[6–11] .

1.1.4 Laboratory Parameters

Data to Laboratory Parameters were obtained from morbidity database including all in-patient stays at all public hospitals of Government Order n.º 254/2018, of September 7th, specifying the price lists to be practiced by the Portuguese NHS, as well as the respective Regulations[12] .

1.1.5 Diagnostic Procedures

For Diagnostic Procedure, the allocated costs were derived from Government Order n.º 254/2018, of September 7th[12].

1.1.6 Interventional Cardiology Procedures costs: Coronary Angiography, Reperfusion Strategy, Coronary Angioplasty and Coronary Artery Bypass Graft (CABG)

1.1.7 Coronary Angiography

For Coronary Angiography, the allocated costs were derived from Government Order n.º 254/2018, of September 7th[12].

1.1.8 Reperfusion Strategy

For Reperfusion Strategy, the allocated costs were derived, in addition to the ones previously cited in the Daily impatient and Operating Room cost categories, from Government Order n.º 254/2018, of September 7th, from the different Framework Agreements established by Portugal's Shared Services of the Ministry of Health (SPMS) and official contracts established by NHS institutions and their suppliers[12,13].

1.1.9 Coronary Angioplasty

For Coronary Angioplasty, the allocated costs were derived, in addition to the ones previously cited in the Daily Impatient and Operating Room cost categories, from Government Order n.º 254/2018, of September 7th, from the different Framework Agreements established by SPMS and from real-world published data[12–14].

1.1.10 Coronary Artery Bypass Graft (CABG)

For CABG, the allocated costs were derived from real-world published data[15].

1.1.11 Other Interventions

For Other Interventions, the allocated costs were derived from Government Order n.º 254/2018, of September 7th, from the different Framework Agreements established by SPMS, from real-world published data and official contracts established by NHS institutions and their suppliers[12,16–18].

Pharmacological treatment

All the various drugs and respect posology's, considered for each drug group, were established based on ProACS registries and scientific consultations. These included amiodarone, mineralocorticoid receptor antagonist (spironolactone), calcium channel blockers, anticoagulants (vitamin K antagonists and noval oral anticoagulants), oral antidiabetic agents, angiotensin converting enzyme inhibitors, aspirin, beta-adrenergic blockers, bivalirudin, angiotensin receptor blockers, clopidogrel, digoxin, diuretics, enoxaparin, fondaparinux, hypolipidemic agents, unfractionated heparin, angiotensin converting enzyme inhibitors, glycoprotein IIb/IIIa inhibitors, sodium-glucose co-transporter inhibitors, inotropic agents, insulin, ivabradine, levosimendan, nitrate/nitrite-like medications, prasugrel, and ticagrelor. Data on the unit costs of pharmaceuticals administered during hospitalisation were obtained from different Framework Agreements established by Shared Services of the Ministry of Health, EPE (SPMS)[19–22].

1.1.12 Complications

For Complications, values were estimated by the mean price of DRG hospitalisation (by severity level), from Government Order n.º 254/2018, of September 7th[12].

1.1.13 Rehabilitation

The costs for Individual Rehabilitation were obtained from Government Order n.º 254/2018, of September 7th[12].

1.1.14 Death

The death costs were calculated by using the total number of cases coded in DRGs (from 2013 to 2018) as AMI of each severity level, which were multiplied by the respective price of hospitalisation, according to Government Order n.º 254/2018, of September 7th, and finally calculated by the average of the sum of the total costs for all severities[12].

1.2 Calculation formulas

$$\begin{aligned}\textbf{Total Individual Cost} \\ &= \text{daily inpatient} + \text{operating room} + \text{medical transport} \\ &+ \text{pharmaceuticals} + \text{laboratory parameters} \\ &+ \text{diagnostic procedure} + \text{coronary angiography} \\ &+ \text{reperfusion strategy} + \text{coronary angioplasty} + \text{CABG} \\ &+ \text{other interventions} + \text{complications} + \text{rehabilitation} + \text{death}\end{aligned}$$

$$\begin{aligned}\textbf{Total Annual ACS Cost} \\ &= [\text{average annual AMI cost} \times \text{total AMI hospitalisations (NHS)}] \\ &+ [\text{average annual UA cost} \times \text{total UA hospitalisations (NHS)}]\end{aligned}$$

Annual percentage burden of the total cost of ACS on the current health expenditure of the Portuguese National and Regional Health Service

$$= \frac{\text{Total annual ACS cost}}{\text{Annual current health expenditure on the Portuguese National and Reginal Health Service}}$$

2 Supplementary Figures and Tables

Supplementary Table 1 Resources used and unit costs (in euros, €)

**Cost category (and components)**	
**Daily inpatient**	**Unit cost (€)**
Total cost (per day)	146.238 €
Hospitality costs	86.098 €
Nurses' salary costs (per hour)	14.200 €
Physicians' salary costs (per hour)	17.540 €
**Operating room**	**Unit cost (€)**
Total cost (per operation)	245.101 €
Standardized occupancy cost	144.911 €
Senior haemodynamic laboratory physicians' salary costs (per hour)	26.560 €
Nurses' salary costs (per hour)	15.230 €
Diagnostic and therapeutic superior technicians' salary costs (per hour)	15.920 €
**Medical transport**	**Unit cost (€)**
Ambulance without a doctor	83.520 €
VMER	49.800 €
Other	66.660 €

Hospital admission location: emergency department	89.106 €
Hospital admission location: intensive care/cardiac intermediate unit	116.240 €
Hospital admission location: haemodynamic laboratory	116.240 €
Hospital admission location: cardiology ward	52.898 €
Hospital admission location: other	52.898 €

**Laboratory Parameters**	**Unit cost (€)**
Biomarkers of myocardial injury measurement	11.388 €
Isolated troponin elevation measurement	9.644 €
Creatinine measurement	1.231 €
Glucose measurement	4.104 €
Haemoglobin measurement	2.667 €
Lipid profile measurement	7.489 €
Troponin assay	9.644 €
Hba1c measurement	7.489 €
Platelet count measurement	2.565 €
BNP measurement	30.367 €
NT-proBNP measurement	30.367 €
Total cholesterol measurement	1.334 €
HDL cholesterol measurement	1.949 €
LDL cholesterol measurement	2.462 €
Triglycerides measurement	1.744 €
Apolipoprotein A measurement	3.078 €
Apolipoprotein B measurement	3.078 €

**Drug group**	**Drugs and respect posology's**	**Source**	**Unit price**	**Drug daily price**	**Average daily cost of drug group**
Amiodarone	amiodarone 200mg once daily	[20]	0.078 €	0.078 €	0.078 €
Aldosterone antagonists	spironolactone 50mg once daily	[20]	0.137 €	0.137 €	0.137 €
Calcium channel blockers	amlodipine 5mg once daily	[20]	0.085 €	0.085 €	
	amlodipine 10mg once daily	[20]	0.128 €	0.128 €	
	lercanidipine 10mg twice daily	[20]	0.104 €	0.207 €	
	lercanidipine 20mg twice daily	[20]	0.143 €	0.286 €	0.177 €
Direct Oral Anticoagulants	apixaban 5mg twice daily	[19]	0.946 €	1.892 €	
	edoxaban 60mg once daily	[19]	2.114 €	2.114 €	
	rivaroxaban 60mg once daily	[19]	4.957 €	4.957 €	2.988 €
	dabigatran 110mg twice daily	[19]	0.888 €	1.776 €	
	dabigatran 150mg twice daily	[19]	0.979 €	1.957 €	1.867 €
Vitamin K antagonists	acenocumarol 4mg once daily	[19]	0.175 €	0.175 €	
	warfarin 5mg once daily	[19]	0.093 €	0.093 €	0.134 €
Oral antidiabetic agents	glucagon-like peptide-1 (GLP-1) analogue liraglutide 0.6mg once daily	[21]	4.211 €	4.211 €	
	alphaglycosidase inhibitor (acarbose) 50mg thrice daily	[21]	0.075 €	0.226 €	
	dipeptidyl peptidyl peptidase-4 (DPP-4) inhibitor alogliptin 25mg once daily	[21]	0.953 €	0.953 €	
	metformin 850mg twice daily	[21]	0.035 €	0.070 €	
	sulphonylurea (gliclazide) 30mg once daily	[21]	0.066 €	0.066 €	
	thiazolidinedione (pioglitazone) 15mg once daily	[21]	0.244 €	0.244 €	0.962 €
Angiotensin receptor antagonists	losartan 50mg once daily	[20]	0.160 €	0.160 €	
	valsartan 80mg once daily	[20]	0.181 €	0.181 €	0.171 €
Aspirin	aspirin 100mg once daily	[20]	0.089 €	0.089 €	0.089 €
Beta-adrenergic blockers	bisoprolol 5mg once daily	[20]	0.047 €	0.047 €	
	carvedilol 25mg twice daily	[20]	0.135 €	0.270 €	0.159 €
Bivalirudin	bivalirudin (250mg) unique dose (STEMI: initial bolus dosage of 0.75mg/kg followed by an infusion rate of 1.75mg/kg/h during coronary angioplasty, with subsequent infusion of 0.25mg/kg/h over 8 hours; NSTEMI/UA: initial bolus dosage of 0.1mg/kg followed	[19]	279.346 €	964.060€ (STEMI) 1514.370€ (NSTEMI/UA)	1239.215 €

	by an infusion rate of 0.25mg/kg/h over 72 hours)				
	clopidogrel 75mg once daily	19	0.252 €	0.252 €	0.252 €
Clopidogrel	clopidogrel 600mg unique load dose	19	2.019 €	2.019 €	2.019 €
Digoxin	digoxin 0.125mg once daily	20	0.028 €	0.028 €	0.028 €
Diuretics	furosemide 40mg once daily	20	0.042 €	0.042 €	0.042 €
	exoparin (40mg/0.4ml) 140mg once daily	19	2.724 €	9.540 €	
	exoparin (60mg/0.6ml) 140mg once daily	19	3.751 €	8.750 €	
	exoparin (80mg/0.8ml) 140mg once daily	19	4.669 €	8.170 €	
Exoparin	exoparin (100mg/1ml) 140mg once daily	19	5.693 €	7.950 €	8.603 €
Fondaparinux	fondaparinux 2.5mg once daily	19	4.084 €	4.084 €	4.084 €
Unfractionated heparin	unfractionated heparin 5000 units unique dose	19	0.924 €	0.924 €	0.924 €
Angiotensin converting enzyme inhibitors	enalapril 20mg twice daily	20	0.103 €	0.206 €	
	lisinopril 20mg twice daily	20	0.185 €	0.370 €	0.288 €
	eptifibatide (75mg/100ml) initial dose of 180 mcg/kg followed by a continuous infusion of 2.0 mcg/kg/min over 24 hours	19	61.320 €	11.221 €	11.221 €
	tirofiban (0.05 mg/mL) initial dose of 25 mcg/kg IV over 5 min followed by a continuous infusion of 0.15 mcg/kg/min over 18 hours	19	248.199 €	258.427 €	258.427 €
Glycoprotein iib/iiia inhibitors	abciximab (10ml/5ml) initial dose of 0.25 mg/kg followed by a continuous infusion of 0.125mcg/kg/min over 12 hours.	22	249.260€	484.295 €	484.295 €
	dobutamine 2 infusion ampoules (250mg each) unique dose	20	2.128 €	4.257 €	
Inotropic agents	dopamine 2 infusion ampoules (250mg each) unique dose	20	1.068 €	2.136 €	3.196 €
Insulin	15 units (each a 3ml cartridge i.e., with 300 IU) once daily	Provided by hospital	4.760 €	71.400 €	71.400 €
	5mg twice daily	20	0.293 €	0.585 €	
Ivabradine	7.5mg twice daily	20	0.226 €	0.451 €	0.518 €
Levosimendan	1 ampoule infusion unique dose	20	699.665 €	699.665 €	699.665 €
	isosorbide dinitrate 20mg twice daily	20	0.049 €	0.098 €	
	Isoxorbide mononitrate 50mg once daily	20	0.133 €	0.133 €	
Nitrate/nitrite-like medications	transdermal nitrate (nitroglycerin) 5mg once daily	20	0.266 €	0.266 €	0.166 €
	PCSK9 inhibitors (evolocumab)	20	200.023 €	200.023 €	
	atorvastatin 40mg once daily	20	0.296 €	0.296 €	
	ezetimibe 10mg once daily	20	0.815 €	0.815 €	
Lipid-lowering agents	rosuvastatin 20mg once daily	20	0.380 €	0.380 €	50.379 €
	10mg once daily	19	1.412 €	1.412 €	1.412 €
Prasugrel	60mg unique load dose	19	8.473 €	8.473 €	8.473 €
	90mg twice daily	19	1.046 €	2.092 €	2.092 €
Ticagrelor	180mg unique load dose	19	2.092 €	2.092 €	2.092 €

**Diagnostic Procedures**	**Unit costs (€)**
12-lead electrocardiogram	6.668 €
Doppler echocardiogram	54.579 €
Exercise stress test	32.932 €
Myocardial perfusion scintigraphy (under physical/pharmacological stress and at rest)	366.203 €
Cardiac computed tomography angiography	216.162 €
Cardiac magnetic resonance imaging (including both morphological and functional assessments)	262.431 €

**Coronary Angiography**	**Unit cost (€)**
Coronary angiography	546.264 €

**Reperfusion Strategy**	**Unit cost (€)**
Fibrinolysis	91.204 €

Primary angioplasty (includes daily impatient and operating room costs)	2,759.779 €

**Coronary Angioplasty**	**Unit cost (€)**
Coronary Angioplasty (bare metal stent)	3,313.467 €
Coronary Angioplasty (drug-eluting stent)	3,514.809 €
Coronary Angioplasty (balloon catheter)	3,254.779 €
Coronary Angioplasty (other)	3,361.018 €
Thrombectomy	377.026 €

**Coronary Artery Bypass Graft (CABG)**	**Unit cost (€)**
Coronary artery bypass graft (CABG)	6,719.026 €

**Other Interventions**	**Unit cost (€)**
Cardiac Resynchronization Therapy (CRT) device	19,609.176 €
Implantable cardioverter defibrillator (ICD)	14,679.176 €
ICD+CRT	19,609.176 €
Swan-Ganz	610.524 €
Intra-aortic balloon	2,177.191 €
Invasive mechanical ventilation	38,667.462 €
Non-invasive mechanical ventilation	3,148.011 €
Permanent pacemaker	1,368.176 €
Temporary pacemaker	1,254.853 €
Ventricular assistance	108,138.388 €

**Complications**	**Unit cost (€)**
Reinfarction	3,505.216 €
Heart failure	2,923.139 €
Mechanical complications	3,721.645 €
Sustained ventricular tachycardia	117.058 €
Resuscitated cardiac arrest	117.058 €
Stroke	4,363.318 €
Major bleeding	6,150.618 €

**Cardiac rehabilitation**	**Unit cost (€)**
Cardiac rehabilitation	32.727 €

**Death**	**Unit cost (€)**
Death	4,001.637 €

Supplementary Table 2 Model Comparison for AIC and Residual Deviance

**Model**	**AIC**	**Residual Deviance**
Gaussian (Identity Link)	1,208,546	2.763×10^{12}
Gaussian (Log Link)	1,208,102	2.7423×10^{12}
Gamma (Identity Link)	-	-
Gamma (Log Link)	1,132,250	39,255

Supplementary Table 3 Annual relative frequencies (in %) of ProACS patient characteristics by admission diagnosis, sex, vital status upon hospital discharge, age group and region

	Admission Diagnosis				Sex		Vital Status		Age group			Regions				
**Year**	**STEMI**	**NSTEMI**	**UA**	**Undetermined AMI**	**Female**	**Male**	**Alive**	**Dead**	**< 55**	**55-74**	**> 75**	**North region**	**Center region**	**Lisbon and Tagus Valley region**	**Alentejo region and Algarve region**	**Autonomous region of the Azores and Autonomous region of Madeira**
2002	42.79%	38.07%	15.85%	3.29%	30.66%	69.34%	93.21%	6.79%	21.74%	50.82%	27.44%	35.51%	20.51%	28.83%	6.36%	8.80%
2003	43.95%	39.91%	12.85%	3.30%	31.26%	68.74%	95.10%	4.90%	22.04%	50.05%	27.90%	31.65%	23.06%	27.03%	9.14%	9.12%
2004	40.62%	42.95%	12.58%	3.84%	29.49%	70.51%	94.82%	5.18%	21.00%	49.86%	29.14%	29.18%	25.64%	24.46%	8.82%	11.90%
2005	41.74%	43.08%	11.31%	3.88%	30.34%	69.66%	95.04%	4.96%	19.52%	51.99%	28.48%	29.01%	23.72%	21.70%	9.59%	15.98%
2006	43.09%	42.27%	10.94%	3.70%	30.45%	69.55%	94.77%	5.23%	20.84%	48.50%	30.65%	29.02%	18.43%	25.99%	8.46%	18.10%
2007	41.71%	44.23%	10.64%	3.42%	29.53%	70.47%	95.21%	4.79%	21.05%	48.61%	30.34%	34.79%	13.29%	24.74%	4.57%	22.61%
2008	46.23%	42.20%	6.79%	4.78%	27.75%	72.25%	95.68%	4.32%	21.76%	46.63%	31.61%	31.32%	17.27%	13.64%	5.47%	32.30%
2009	45.84%	43.40%	7.56%	3.20%	30.11%	69.89%	94.63%	5.37%	21.59%	48.56%	29.85%	28.16%	26.27%	14.75%	2.49%	28.33%
2010	44.38%	44.95%	8.07%	2.60%	28.36%	71.64%	96.15%	3.85%	21.83%	47.90%	30.26%	29.08%	33.36%	12.11%	1.59%	23.86%
2011	45.68%	40.48%	10.71%	3.13%	29.48%	70.52%	95.63%	4.37%	21.90%	47.95%	30.15%	25.27%	42.84%	12.93%	4.41%	14.56%
2012	40.29%	45.22%	11.48%	3.01%	29.52%	70.48%	95.41%	4.59%	20.10%	48.42%	31.48%	16.53%	43.25%	19.77%	4.72%	15.73%
2013	38.58%	46.75%	11.79%	2.87%	29.37%	70.63%	96.23%	3.77%	21.37%	47.95%	30.68%	17.64%	43.27%	18.56%	4.26%	16.27%
2014	39.53%	46.74%	11.11%	2.62%	27.26%	72.74%	96.38%	3.62%	19.74%	49.16%	31.10%	18.26%	47.32%	14.03%	3.67%	16.72%
2015	45.18%	44.81%	7.61%	2.40%	25.95%	74.05%	97.17%	2.83%	20.57%	49.45%	29.98%	20.65%	41.46%	18.79%	2.65%	16.45%
2016	44.15%	47.60%	5.92%	2.33%	25.75%	74.25%	96.32%	3.68%	21.26%	49.51%	29.23%	20.68%	38.41%	21.46%	1.49%	17.97%
2017	47.06%	46.18%	4.22%	2.53%	27.54%	72.46%	96.39%	3.62%	20.73%	50.80%	28.47%	8.27%	30.97%	34.22%	2.53%	24.01%
2018	49.77%	42.84%	5.08%	2.31%	26.44%	73.56%	95.67%	4.33%	19.28%	52.62%	28.10%	6.79%	40.92%	19.02%	2.72%	30.55%
2019	44.64%	45.76%	5.91%	3.68%	27.53%	72.47%	96.98%	3.02%	20.14%	52.09%	27.77%	3.03%	46.59%	19.21%	4.13%	27.04%
2020	48.53%	43.78%	4.41%	3.28%	26.95%	73.05%	96.39%	3.61%	23.74%	48.59%	27.67%	8.49%	32.05%	24.55%	0.86%	34.04%
2021	48.17%	45.25%	4.23%	2.35%	26.35%	73.65%	98.05%	1.95%	22.88%	48.56%	28.56%	11.72%	22.14%	57.30%	0.09%	8.74%
2022	54.51%	38.18%	6.75%	0.55%	28.56%	71.44%	97.14%	2.86%	20.00%	50.75%	29.25%	17.95%	22.56%	59.28%	0.00%	0.21%

Supplementary Table 4 Relative frequency (in %) of each cost category by overall, year, admission diagnosis, sex, vital status upon hospital discharge and age group

	Daily inpatient	Medical transport	Laboratory Parameters	Diagnostic Procedures	Coronary angiography	Reperfusion Strategy	Coronary Angioplasty	CABG	Other Interventions	Pharmacological treatment	Complications	Rehabilitation	Death
**Overall**													
	16.69	0.51	5.33	0.52	6.75	8.85	31.47	0.97	13.85	2.07	10.08	0.05	2.87
**Year**													
2002	22.28	0.00	4.11	0.00	4.72	3.91	20.16	2.54	22.46	0.91	14.18	0.00	4.72
2003	23.84	0.00	4.72	0.00	5.55	4.07	26.10	1.59	14.21	1.09	15.02	0.00	3.82
2004	22.80	0.00	4.68	0.00	6.19	4.49	30.85	1.19	12.05	1.15	12.71	0.00	3.89
2005	18.41	0.00	4.05	0.00	6.12	4.96	33.77	1.75	13.84	1.08	12.85	0.00	3.18
2006	16.86	0.00	3.76	0.00	6.03	5.78	37.52	0.85	14.09	0.99	11.04	0.00	3.08
2007	15.67	0.00	3.79	0.00	6.64	7.09	38.65	1.26	11.36	0.92	11.79	0.00	2.83
2008	12.86	0.00	3.61	0.00	6.16	9.92	42.37	0.62	10.04	0.72	11.38	0.00	2.33
2009	12.73	0.00	3.41	0.01	5.98	9.82	40.64	0.52	13.03	0.67	10.43	0.00	2.75
2010	12.50	0.46	4.88	0.46	6.41	10.38	39.30	0.54	10.65	1.73	10.58	0.03	2.07
2011	11.15	0.76	5.51	0.80	6.09	9.55	36.15	1.21	15.48	2.83	8.22	0.07	2.18
2012	11.68	0.77	5.86	0.82	6.35	10.35	37.54	0.26	12.92	2.82	8.15	0.06	2.43
2013	11.83	0.87	6.29	0.94	6.92	11.12	39.25	0.29	9.54	2.89	7.84	0.07	2.15
2014	12.04	0.81	5.98	0.91	6.48	10.29	38.98	0.11	11.65	3.17	7.52	0.06	1.99
2015	12.38	0.79	6.08	0.90	6.96	10.84	41.11	0.20	8.98	2.94	7.18	0.06	1.58
2016	13.07	0.80	6.02	0.89	6.77	10.07	39.61	0.09	10.18	3.06	7.35	0.08	2.01
2017	13.41	0.83	5.71	0.85	6.81	11.15	38.63	0.53	11.66	2.56	5.80	0.09	1.96
2018	16.17	1.02	4.95	0.86	7.82	10.34	37.05	0.62	10.70	2.12	5.72	0.14	2.49
2019	17.00	1.14	4.69	0.86	6.94	9.39	41.26	0.76	9.17	2.25	4.55	0.09	1.91
2020	12.88	0.87	4.38	0.74	6.75	9.34	42.77	0.27	13.52	2.11	4.46	0.12	1.80
2021	17.54	0.92	5.55	0.75	8.39	8.55	46.11	0.30	4.65	2.19	3.73	0.04	1.27
2022	21.56	0.51	6.07	0.69	7.80	7.45	34.55	1.47	13.70	1.08	2.73	0.10	2.30
**Admission Diagnosis**													
STEMI	12.80	0.38	4.43	0.40	5.59	15.57	30.60	0.44	16.09	1.42	9.02	0.04	3.24
NSTEMI	21.01	0.69	7.17	0.68	7.99	0.44	32.83	1.38	10.87	2.87	11.58	0.06	2.43
UA	26.15	0.58	2.58	0.75	11.27	0.63	39.98	3.55	4.47	3.20	6.09	0.03	0.73
Undetermined AMI	18.49	0.49	5.32	0.45	4.97	1.25	18.11	1.07	24.52	2.58	18.43	0.04	4.28
**Sex**													
Female	17.67	0.49	5.29	0.50	6.13	7.15	26.30	0.83	15.34	2.26	13.79	0.04	4.20
Male	16.27	0.52	5.35	0.53	7.00	9.54	33.57	1.02	13.26	1.99	8.59	0.05	2.33
**Vital Status**													
Alive	18.44	0.56	5.85	0.58	7.57	9.75	35.29	1.09	9.33	2.26	9.23	0.05	0.00
Dead	4.91	0.13	1.85	0.10	1.21	2.79	5.73	0.11	44.37	0.77	15.84	0.00	22.19
**Age Group**													
< 55	14.48	0.52	5.51	0.54	7.83	12.63	39.10	0.78	12.06	1.47	4.35	0.06	0.68
55-75	16.54	0.51	5.23	0.52	7.09	8.94	33.17	1.22	14.19	2.08	8.52	0.05	1.95
> 75	18.45	0.50	5.40	0.51	5.44	6.11	23.45	0.66	14.39	2.47	16.66	0.04	5.91

Supplementary Table 5 Relative frequency (in %) of each component in the other interventions category cost between 2002 and 2022

Year	CRT	ICD	ICD+CRT	Swan-Ganz	Intra-aortic balloon	Invasive mechanical ventilation	Non-invasive mechanical ventilation	Permanent pacemaker	Temporary pacemaker	Ventricular assistance
2002	-	-	-	0.04	0.44	22.60	-	-	-	-
2003	-	-	-	0.00	0.06	14.90	-	-	-	-
2004	-	-	-	0.02	0.13	12.66	-	-	-	-
2005	-	-	-	0.02	0.13	14.62	-	-	-	-
2006	-	-	-	-	0.20	14.97	-	-	-	-
2007	-	-	-	0.01	0.15	12.16	-	-	-	-
2008	-	-	-	0.01	0.15	10.93	-	-	-	-
2009	-	-	-	-	0.25	13.92	-	-	-	-
2010	-	0.18	-	0.00	0.29	10.42	0.37	0.05	0.16	-
2011	-	0.14	0.06	0.02	0.36	13.12	0.84	0.10	0.39	1.03
2012	-	0.21	0.07	0.02	0.22	11.61	0.92	0.12	0.32	-
2013	0.08	0.12	0.24	0.05	0.14	8.34	0.64	0.10	0.27	-
2014	-	0.20	-	0.05	0.10	10.48	0.64	0.08	0.25	0.48
2015	-	0.20	-	0.01	0.08	8.13	0.78	0.08	0.19	-
2016	-	0.43	-	0.01	0.08	9.43	0.60	0.04	0.18	-
2017	-	0.45	-	0.00	0.08	11.12	0.64	0.09	0.15	-
2018	-	0.34	0.31	-	0.10	9.98	0.79	0.04	0.15	-
2019	-	0.49	-	-	0.02	8.41	1.00	0.08	0.10	-
2020	0.18	0.13	0.36	-	0.04	13.03	1.00	0.12	0.17	-
2021	0.73	1.09	1.10	0.01	-	2.16	0.29	0.20	0.14	-
2022	0.32	1.22	0.97	-	0.11	5.77	0.42	0.39	0.21	7.17

118 **Supplementary Table 6** Results of sensitivity analyses

Resource parameter	Interval in mean cost for the decrease and increase simulation (in euros)	Percentage change interval in mean cost for the increase and decrease scenarios (in %)
Daily inpatient	[6068.74; 6284.79]	[-3.38; 0.06]
Medical transport	[6274.45; 6291]	[-0.1; 0.16]
Biomarkers of myocardial injury measurement	[6233.67; 6289.22]	[-0.75; 0.13]
Isolated troponin elevation measurement	[6279.66; 6287.38]	[-0.02; 0.1]
Creatinine measurement	[6278.97; 6282.69]	[-0.03; 0.03]
Glucose measurement	[6275.04; 6287.35]	[-0.09; 0.1]
Haemoglobin measurement	[6276.75; 6284.75]	[-0.06; 0.06]
Lipid profile measurement	[6280.3; 6281.8]	[-0.01; 0.02]
Troponin assay	[6280.74; 6282.67]	[0; 0.03]
Hba1c measurement	[6280.38; 6281.88]	[-0.01; 0.02]
Platelet count measurement	[6280.54; 6281.06]	[0; 0]
BNP measurement	[6278.15; 6290.3]	[-0.04; 0.15]
NT-proBNP measurement	[6278.36; 6290.51]	[-0.04; 0.15]
Total cholesterol measurement	[6280.67; 6280.94]	[0; 0]
HDL cholesterol measurement	[6280.62; 6281.01]	[0; 0]
LDL cholesterol measurement	[6280.58; 6281.08]	[0; 0]
Triglycerides measurement	[6280.64; 6280.99]	[0; 0]
Apolipoprotein A measurement	[6280.67; 6281.29]	[0; 0.01]
Apolipoprotein B measurement	[6280.67; 6281.28]	[0; 0.01]
Total medication	[6254.64; 6283.12]	[-0.42; 0.04]
Reperfusion strategy	[6171.03; 6555.14]	[-1.75; 4.37]
Coronary Angioplasty	[6199.94; 6309.2]	[-1.29; 0.45]
Coronary Angioplasty (aditional procedure)	[6276.84; 6386.09]	[-0.06; 1.68]
coronary angioplasty (left main artery)	[6274.82; 6966.16]	[-0.09; 10.91]
coronary angioplasty (left anterior descending artery)	[6111.54; 6797.59]	[-2.69; 8.23]
coronary angioplasty (circumflex artery)	[6191.17; 6874.78]	[-1.43; 9.46]
coronary angioplasty (right coronary artery)	[6157.71; 6840.12]	[-1.96; 8.91]
coronary angioplasty (bypass as an additional procedure)	[6276.64; 6956.14]	[-0.07; 10.75]
Thrombectomy	[6277.37; 6352.78]	[-0.05; 1.15]
Coronary artery bypass graft (CABG)	[6268.6; 7605.54]	[-0.19; 21.09]
12-lead electrocardiogram + Doppler echocardiogram	[6274.59; 6288.17]	[-0.1; 0.12]
Exercise stress test	[6280.77; 6287.36]	[0; 0.1]
Myocardial perfusion scintigraphy (under physical/pharmacological stress and at rest)	[6280.58; 6353.82]	[0; 1.16]
Cardiac computed tomography angiography	[6280.75; 6323.98]	[0; 0.69]
Cardiac magnetic resonance imaging (including both morphological and functional assessments)	[6280.74; 6333.22]	[0; 0.83]
Cardiac Resynchronization Therapy (CRT) device	[6280.47; 10202.31]	[-0.01; 62.44]
Implantable cardioverter defibrillator (ICD)	[6278.64; 9214.47]	[-0.03; 46.71]
ICD+CRT	[6279.83; 10201.67]	[-0.02; 62.43]
Swan-Ganz	[6280.57; 6402.68]	[0; 1.94]
Intra-aortic balloon	[6278.52; 6713.96]	[-0.04; 6.9]
Invasive mechanical ventilation	[6123.45; 13856.94]	[-2.51; 120.62]

Non-invasive mechanical ventilation	[6275.46; 6905.06]	[-0.08; 9.94]
Permanent pacemaker	[6280.08; 6553.71]	[-0.01; 4.35]
Temporary pacemaker	[6279.05; 6530.02]	[-0.03; 3.97]
Ventricular assistance	[6277.27; 27904.95]	[-0.06; 344.29]
Reinfarction	[6272.97; 6974.02]	[-0.12; 11.04]
Heart failure	[6178.74; 6763.36]	[-1.62; 7.68]
Mechanical complications	[6274.31; 7018.64]	[-0.1; 11.75]
Sustained ventricular tachycardia	[6280.63; 6304.04]	[0; 0.37]
Resuscitated cardiac arrest	[6280.21; 6303.62]	[-0.01; 0.36]
Stroke	[6274.68; 7147.35]	[-0.1; 13.8]
Major bleeding	[6277.39; 7507.51]	[-0.05; 19.53]
Cardiac rehabilitation	[6280.19; 6286.74]	[-0.01; 0.09]
Death	[6244.77; 7045.1]	[-0.57; 12.17]

3 Supplementary Figures

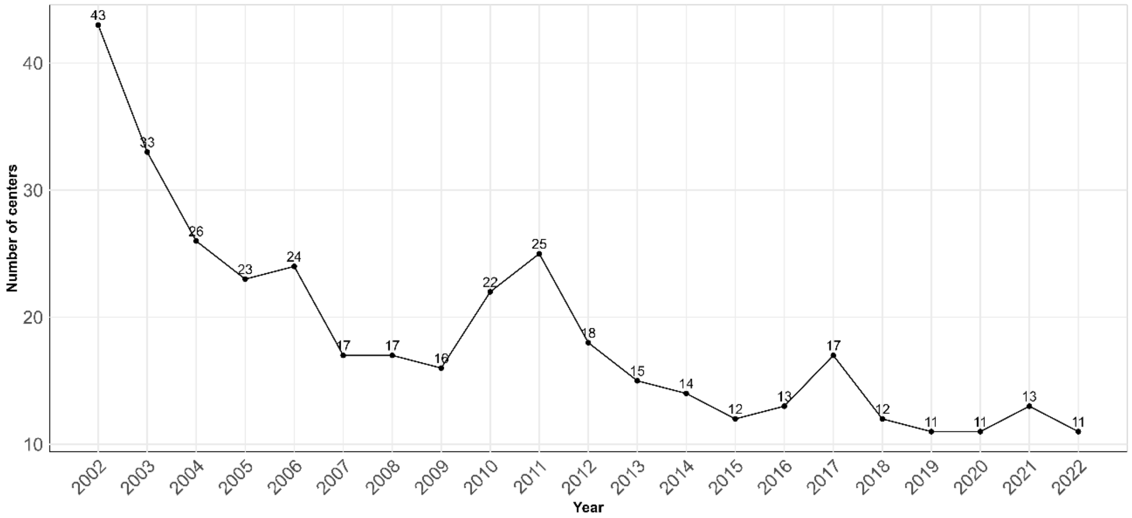

Supplementary Figure 1 Evolution of the annual number of centers in ProACS

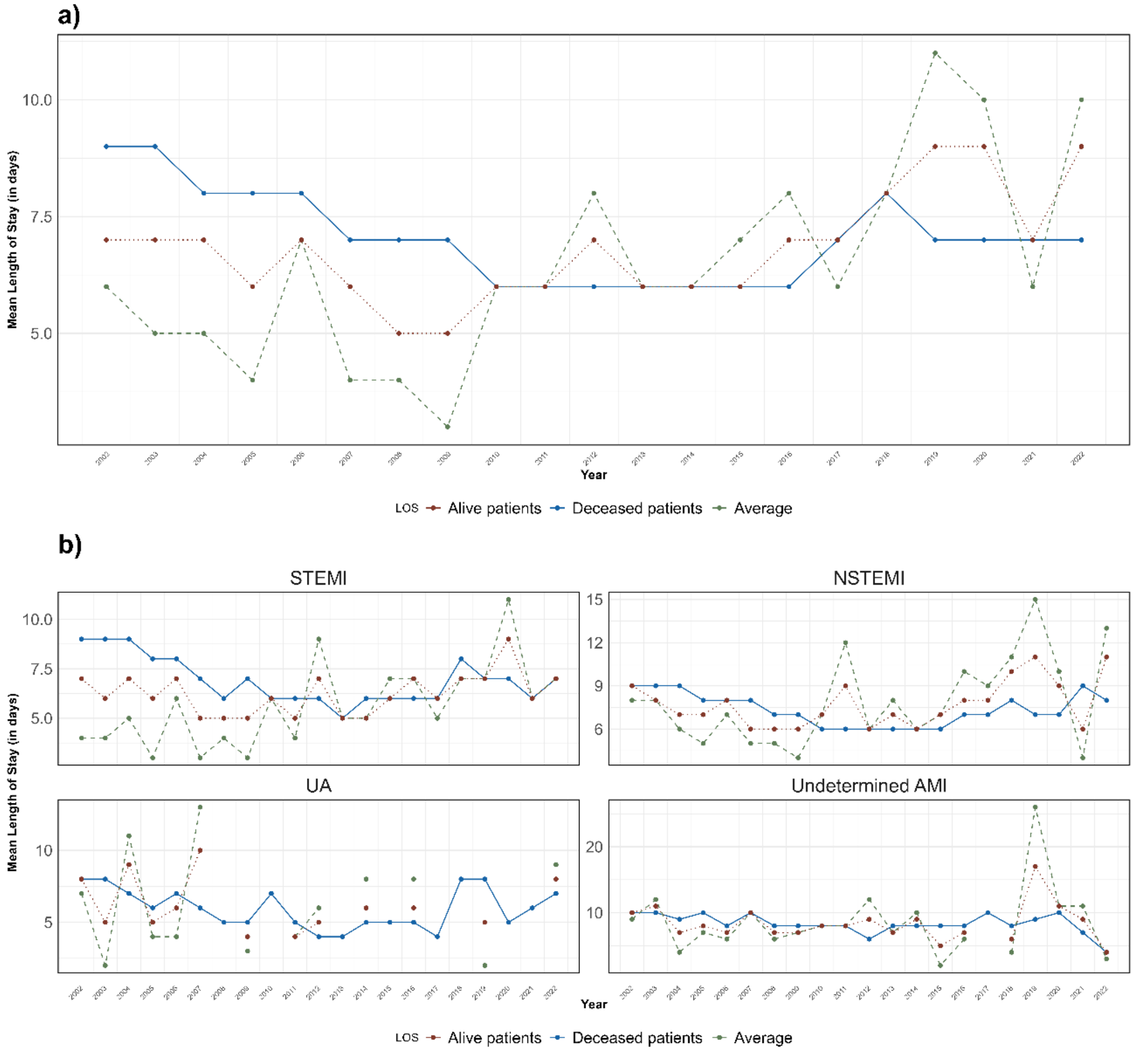

Supplementary Figure 1 Average annual LOS in the hospital by admission year (a) and by admission year and diagnosis (b), for alive and deceased patients (and their average)

4 References

1. SIM. Regimes de trabalho: tabela salarial dos médicos do SNS [Internet]. Lisboa: Sindicato Independente dos Médicos; 2022 [cited 2023 Dec 18]. Available from: https://www.simedicos.pt/fotos/editor2/ficheiros/tabela_salarial_2022.pdf

2. SEP. Tabela Salarial Carreira Especial Enfermagem 2022 [Internet]. Lisboa: Sindicato dos Enfermeiros Portugueses; 2022 [cited 2023 Dec 18]. Available from: https://www.sep.org.pt/files/uploads/2017/06/sep_14122021_Tabela_salarial_Enfermagem_2022vhora-TExtrord.pdf

3. Ferreira JP, Araújo F, Dores J, Santos L, Pape E, Reis M, et al. Hospitalization costs due to hypoglycemia in patients with diabetes: a microcosting approach. Diabetes Ther. 2020;11(10):2237–55.

4. STSS. Tabela Salarial 2022: Carreira Especial Técnico Superior de Diagnóstico e Terapêutica [Internet]. Lisboa: Sindicato Nacional dos Técnicos Superiores de Saúde nas áreas de Diagnóstico e Terapêutica; 2022 [cited 2023 Dec 18]. Available from: https://www.stss.pt/images/_Data/Legislacao/STSS_-_Tabela_Salarial_TSDT_2022.pdf

5. Portugal. Ministério da Saúde. Avaliação da situação nacional dos blocos operatórios: relatório final. Lisboa; 2015.

6. INEM. Relatório de atividade dos meios de emergência médica 2022. Lisboa: Instituto Nacional de Emergência Médica; 2022.

7. Despacho n.º 1858/2016. Diário da República n.º 25/2016, Série II de 2016-02-05. 4494-4495.

8. Despacho n.º 14898/2011. Diário da República n.º 211/2011, Série II de 2011-11-03. 43563-43564.

9. Despacho n.º 5561/2014. Diário da República n.º 79/2014, Série II de 2014-04-23. 11123-11124.

10. Tribunal de Contas. Auditoria de resultados ao Instituto Nacional de Emergência Médica: relatório de auditoria nº 47/2010 2ªS. Lisboa: Tribunal de Contas; 2010.

11. Portaria n.º 207/2017. Diário da República n.º 132/2017, Série I de 2017-07-11. 3550-3708.

12. Portaria n.º 254/2018. Diário da República n.º 173/2018, Série I de 2018-09-07. 4497-4706.

13. Portugal. Ministério da Saúde. SPMS. Caderno de encargos: Acordo quadro para fornecimento de stents coronários às instituições e serviços do Serviço Nacional de Saúde: CP 2020/34. Lisboa: Serviços Partilhados do Ministério da Saúde; 2021.

14. Portugal. Ministério da Saúde. CHULC. Contrato nº 470/2023: Aquisição de cateteres de balão e fios guia para angioplastia coronária ao CHULC, EPE. Lisboa: Centro Hospitalar Universitário de Lisboa Central; 2023.

15. Lima AM da S. Avaliação económica das intervenções de cirurgia cardíaca realizadas num Hospital do SNS [Dissertação de mestrado]. Porto: Faculdade de Economia. Universidade do Porto; 2019.

16. Portugal. Ministério da Saúde. SPMS. Caderno de encargos: Acordo quadro para fornecimento de cardioversores desfibrilhadores implantáveis e pacemakers na área da saúde: CP 2021/33. Lisboa: Serviços Partilhados do Ministério da Saúde; 2021.

17. Schueler S, Silvestry SC, Cotts WG, Slaughter MS, Levy WC, Cheng RK, et al. Cost-effectiveness of left ventricular assist devices as destination therapy in the United Kingdom. ESC Heart Fail. 2021;8(4):3049–57.

18. Portugal. Ministério da Saúde. HFF. Caderno de encargos: Ajuste direto nº 11E0063323: Aquisição de cateteres Swan Ganz e adaptadores de sonda, para o Hospital Professor Doutor

Fernando Fonseca, EPE, para o ano de 2023. Lisboa: Hospital Professor Doutor Fernando Fonseca; 2023.

19. Portugal. Ministério da Saúde. SPMS. Caderno de encargos: 304/2023: Acordo quadro para fornecimento de medicamentos do grupo 4: sangue, na área da saúde. Lisboa: Serviços Partilhados do Ministério da Saúde; 2023.

20. Portugal. Ministério da Saúde. SPMS. Caderno de encargos: 305/2023: Acordo quadro para fornecimento de medicamentos do aparelho cardiovascular, na área da saúde. Lisboa: Serviços Partilhados do Ministério da Saúde; 2023.

21. Portugal. Ministério da Saúde. SPMS. Caderno de encargos: Acordo quadro para fornecimento de medicamentos do aparelho locomotor, usados no tratamento das doenças endócrinas e hormonas às instituições e serviços do Serviço Nacional de Saúde: CP 2021/47. Lisboa: Serviços Partilhados do Ministério da Saúde; 2021.

22. Portugal. Ministério da Saúde. SPMS. Caderno de encargos: Acordo quadro para fornecimento de medicamentos do grupo 4: sangue às instituições e serviços do Serviço Nacional de Saúde: CP 2020/18. Lisboa: Serviços Partilhados do Ministério da Saúde; 2020.
